# Penetrating the biofilm barrier: characterization of *Escherichia* phage vB_EcoS-TPF103dw and harnessing depolymerase to combat Shiga toxin-producing *Escherichia coli* O103 biofilm

**DOI:** 10.3389/fmicb.2025.1715907

**Published:** 2025-11-27

**Authors:** David L. Campos, Yen-Te Liao, Leslie A. Harden, Yujie Zhang, Vivian C. H. Wu

**Affiliations:** Produce Safety and Microbiology Research Unit, U.S. Department of Agriculture, Agricultural Research Service, Western Regional Research Center, Albany, CA, United States

**Keywords:** biofilm, STEC, *E. coli* O103, depolymerase, bacteriophage

## Abstract

**Introduction:**

Besides Shiga Toxin-producing *Escherichia coli* (STEC) O157:H7, non-O157 STEC strains, such as O103, have been linked to outbreaks in meat, dairy, and produce. This study aimed to characterize and evaluate the newly isolated *Tequintavirus* phage, vB_EcoS-TPF103dw, as an intervention against STEC O103 biofilm.

**Methods:**

Phage vB_EcoS-TPF103dw isolated from chicken feces, was sequenced and biologically characterized. Antimicrobial activity was tested *in vitro* and against O103 biofilm on stainless steel. Biofilm disruption was examined by scanning electron microscopy (SEM).

**Results:**

TPF103dw, belonging to the *Tequintavirus* genus, has a latent period of approximately 50 min, with an estimated burst size of 232 PFU/cell, and is stable over a wide range of pH (pH 5 to pH 10) and temperature (4 to 60 °C). Phage TPF103dw encoded four high-probability (>90%) depolymerase candidates. The results showed filtrate containing soluble phage-derived enzymes alone were sufficient to dismantle the extracellular polysaccharide layer, as confirmed by SEM. Phage application against STEC O103 biofilm on stainless-steel coupons for 30 min resulted in a significant STEC O103 reduction of 0.83 log CFU/coupon.

**Discussion:**

The findings of this study provide insights into a novel *Tequintavirus* phage, vB_EcoS-TPF103dw, and demonstrate its genomic diversity, predicted depolymerase-encoding potential, stability under variable conditions, and antimicrobial efficacy against STEC O103 biofilms *in vitro*.

## 1 Introduction

Shiga toxin-producing *Escherichia coli* (STEC) serotype O103 has emerged as a growing concern in public health due to its increasing association with severe foodborne illnesses (Nemati et al., 2025). In the United States, O103 has been recognized as one of the six major non-O157 STEC serogroups, collectively responsible for the majority of non-O157 STEC infections (Alharbi et al., 2022). Although STEC O157:H7 remains the most frequently studied serogroup, non-O157 strains such as O103 have gained attention for their involvement in outbreaks linked to contaminated meat (Schimmer et al., 2008), dairy products (Heinsbroek et al., 2025; Mylius et al., 2018), and produce (Smith et al., 2022). STEC O103 demonstrates remarkable resilience not only deriving from its virulence factors but also from its capacity to form robust, persistent biofilms on a variety of food-contact surfaces in food production settings (Nan et al., 2022). These biofilms serve as protective reservoirs that enable bacterial communities to withstand environmental stresses, sanitizers, and conventional antimicrobial agents, making them particularly difficult to eradicate using standard cleaning protocols (Maillard and Centeleghe, 2023; Sanchez-Vizuete et al., 2015; Waters and Hung, 2014).

Biofilms are microbial communities organized into complex, three-dimensional structures stabilized by extracellular polymeric substances (EPS), which contain polysaccharides, proteins, and nucleic acids (Wong et al., 2023). In food production, these EPS-rich matrices shield pathogens, including STEC, complicating sanitation (Sinclair et al., 2024). This matrix provides significant protection to resident bacteria, impedes the penetration of disinfectants, and prevents the cells from both chemical and physical threats (Ban-Cucerzan et al., 2025). The presence of EPS also enhances bacterial adhesion to surfaces (Flemming and Wingender, 2010). Due to these defensive features, the complete removal of biofilm-associated pathogens, including STEC O103, remains a major challenge for food safety and public health systems.

Bacteriophages that infect and lyse bacteria have emerged as promising biological alternatives to chemical sanitizers for disrupting biofilms (Carlton et al., 2005; Jaroni et al., 2023; Mayorga-Ramos et al., 2024; Meneses et al., 2023; Wiguna et al., 2022; Zhang et al., 2024). In addition to their host specificity and self-amplifying nature, many phages produce enzymes known as depolymerases, which are capable of degrading the structural components of bacterial biofilms (Guo et al., 2017, 2023). These depolymerases often reside in the phage’s tail fibers, tail spikes, or baseplate, where they assist in breaching bacterial surface barriers during infection (Yang et al., 2023). In some cases, depolymerases are enclosed in the phage capsid or released as free-floating enzymes following bacterial lysis (Guliy and Evstigneeva, 2025). Their activity facilitates phages’ access to host cells that are deeply embedded within the biofilm, thereby contributing to the enhancement of overall phage application efficacy.

Depolymerases are polysaccharide-degrading enzymes with a wide range of degradation specificities. Depolymerases not only target the EPS of biofilms but can also degrade critical bacterial surface structures such as capsular polysaccharides (CPS) and lipopolysaccharides (LPS) (Knecht et al., 2019; Park and Park, 2021). Capsular depolymerases act as an adjuvant and cleave the long-chain polysaccharides that form the capsule surrounding some bacterial strains, exposing the bacterial surface and making it more accessible to phage infection or host immune mechanisms (Lin et al., 2017; Mushtaq et al., 2005; Yang et al., 2025). LPS-targeting depolymerases degrade the outer leaflet of the Gram-negative outer membrane, destabilizing the bacterial envelope and sensitizing the cell to environmental pressures (Falgenhauer et al., 2019). Their enzymatic specificity and potency make them highly attractive tools for degrading the structural defenses of biofilm-producing and encapsulated bacteria.

Despite increasing interest in the use of phages and depolymerases for biofilm control, relatively few studies have focused on non-O157 STEC serogroups (Liao et al., 2022a; Liao et al., 2019; Park and Park, 2019; Zhang et al., 2021a,b), and even fewer have investigated depolymerase activity specific to STEC O103. Given the variability in capsular and LPS structures among *Escherichia coli* (*E. coli*) serotypes (Chart et al., 1993), it cannot be assumed that depolymerases effective against one group will be effective against another. Therefore, targeted research on depolymerases specific to STEC O103 is essential to develop effective interventions against this pathogen in food processing environments. The objective of this study was to isolate and characterize the novel bacteriophage vB_EcoS-TPF103dw from chicken feces, assess its lytic activity against *E. coli* O103, and evaluate its potential biofilm-disrupting capabilities.

## 2 Methods

### 2.1 Isolation of phage

*Escherichia* phage vB_EcoS-TPF103dw was previously isolated from chicken feces collected at a local regional park using the bacterial host *E. coli* ATCC 13706, following slight modified methods by Liao et al. (2019). In brief, a single generic *E. coli* laboratory host without bacterial pre-enrichment to minimize host selection bias was used in this study, rather than an enrichment-based method using a multi-strain STEC cocktail. To ensure clonality and genetic purity, five sequential rounds of plaque purification were performed, compared to three in the original protocol. For propagation, 50 μL of high-titer phage lysate (approximately 10^9^ PFU/mL) was combined with 45 mL of an overnight culture of *E. coli* ATCC 13706 culture in Tryptic Soy Broth (TSB; Becton Dickinson, Sparks, MD, United States). Calcium Chloride was added to a final concentration of 10 mM to promote infection, and the mixture was incubated at 37 °C for 20 h. After incubation, the phage was centrifuged at 8,000 x g for 10 min and then passed through a 0.22 μm filter membrane to remove bacterial debris for further downstream processing and experimentation.

### 2.2 Bacterial strains

Non-pathogenic *E. coli* ATCC 13706, several Shiga toxin-producing *E. coli* (STEC) serogroups (O26, O45, O103, O111, O121, O145, and O157), *E. albertii*, and *Salmonella enterica* serovars (*Typhimurium, Enteritidis, and Infantis*) were obtained from the Produce Safety and Microbiology Research Unit of the USDA Agricultural Research Service at the Western Regional Research Center (Albany, CA, United States). These bacterial isolates were employed to evaluate the host range and plating efficiency of the phage characterized in this study. The principal host used for phage isolation, *E. coli* ATCC 13706, was further applied for phage propagation, titer determination, and one-step growth experiments. Prior to testing, each strain was cultivated by transferring a loopful of cells into 10 mL of tryptic soy broth (TSB) and incubating at 37 °C for 20 h.

Phage titers were determined using a double-layer plaque assay. Briefly, ten-fold serial dilutions of phage stocks were prepared in SM buffer, mixed with an exponentially growing host culture, combined with molten soft top agar, and overlaid onto tryptic soy agar (TSA; Becton Dickinson, Sparks, MD, United States) base plates. Plates were incubated at 37 °C until discrete plaques were visible. Those yielding 30–300 plaques were counted in duplicate, and the counts were averaged. Phage titer (PFU/mL) was calculated from the averaged counts using the reciprocal dilution and plated volume. All assays were performed on TSA with dilutions in SM buffer, and the working stock titer was confirmed a few days prior to the experiments.

### 2.3 Whole-genome sequencing

Phage TPF103dw was purified through a cesium chloride (CsCl) gradient centrifugation to remove bacterial debris as previously described by Liao et al. (2022b). Phage DNA was extracted using a Norgen phage DNA extraction kit (Thorold, ON, Canada), followed by DNA library preparation before sequencing, as previously described by Zhang et al. (2021a,b), The libraries were loaded onto an Illumina MiSeq using the MiSeq Reagent Kit v2 (500 cycle). In brief, raw reads underwent quality screening with FASTQC (version v0.12.1), trimming in Trimmomatic (version v0.39) at a Q30 threshold, *de novo* assembly with Unicycler (v0.4.8), and gene prediction plus functional annotation with Prokka (v1.14.6) under default parameters (Ramsey et al., 2020; Seemann, 2014). A circular genome map was generated using Proksee software (Grant et al., 2023). The genome sequence of *Escherichia* phage vB_EcoS- TPF103dw was deposited at the National Center for Biotechnology Information (NCBI) database under GenBank accession number # PX092659. The phage genome was screened in Galaxy (Galaxy-Phage) using ABRicate v1.0.1 (Seemann, 2016); database versions matched those referenced by the tool at run time. For antimicrobial resistance determinants, we enabled ARG-ANNOT, CARD, MEGARes, NCBI (AMRFinderPlus), and ResFinder. For virulence factors, we used VFDB and the *E. coli* virulence-factor set (ecoli_vf). We additionally ran ecoh to report *E. coli* O:H serotype markers and PlasmidFinder to identify plasmid replicons.

### 2.4 Comparative genomics

Twenty reference phage genomes with the highest scores, similar to the whole-genome nucleotide sequence of vB_EcoS-TPF103dw, were obtained via BLASTn v2.16.0 search against the NCBI database (Camacho et al., 2009). The whole-genome phylogenetic analysis of phage vB_EcoS-TPF103dw and 20 reference phages was performed using the Virus Classification and Tree Building Online Resource (VICTOR) webserver^1^ (Meier-Kolthoff and Göker, 2017). All pairwise comparisons of the nucleotide sequences were conducted using the Genome-BLAST Distance Phylogeny (GBDP) method (Meier-Kolthoff et al., 2013) under settings recommended for prokaryotic viruses (Meier-Kolthoff and Göker, 2017). Branch support was inferred from 100 pseudo-bootstrap replicates each. Trees were rooted at the midpoint (Farris, 1972) and visualized with ggtree (Yu, 2020). Average nucleotide identity among phage TPF103dw and the selected reference phages was calculated based on BLAST+ (ANIb) using the JSpeciesWS web server (Richter et al., 2016). In addition, Maximum-likelihood phylogenetic analysis was conducted on amino acid sequences of two potential depolymerase enzymes (ORF 17 and ORF 18) and several known depolymerase enzymes from the reference phages with 100 bootstrap replicates (Supplementary Figure S6).

#### 2.4.1 Prediction of depolymerase enzyme

Open reading frames (ORF 17, ORF 18) annotated from the genome of phage vB_EcoS-TPF103dw using Prokka v1.14.6 on the Galaxy phage platform (Ramsey et al., 2020) were screened with DePP v1.0 (Magill and Skvortsov, 2023). Because the DePolymerase Predictor (DePP) outputs a probability score for depolymerase function, no clear threshold is set, and interpretation is at the discretion of the user. Thus, to increase stringency and reduce false positives, we required a DePP score of 0.90 or higher in this study. Both Candidates scored ≥0.90 for predicted depolymerase activity (Supplementary Table S1). The amino acid sequences of these ORFs were queried against the NCBI clustered non-redundant (nr) protein database using BLASTp v2.16.0 (Camacho et al., 2009) with default parameters (BLOSUM62; e-value 0.05). The resulting homologous sequences were visualized with the NCBI “Distance Tree of Results” tool, which applies Grishin protein distances, constructs Neighbor-Joining phylogenies, and annotates branches with percent identity to the query. To further validate predicted functions, the amino acid sequences of these candidate ORFs were analyzed using InterProScan v105.0 (Blum et al., 2025) to identify conserved domains associated with depolymerase activity.

### 2.5 One-step growth curve

A one-step growth curve of phage vB_EcoS-TPF103dw was conducted on *E. coli* ATCC 13706 using previously described methods with slight modifications (Liao et al., 2022b). To initiate the *E. coli* culture, 10 mL of tryptic soy broth (Becton Dickinson, Sparks, MD, United States) was inoculated with *E*. *coli* ATCC 13706 and incubated at 37 °C for 18 to 24 h. The next day, 0.2 mL of the overnight culture was transferred into 19.8 mL of fresh TSB and incubated at 37 °C with shaking (90 rpm) for 2 h to reach the log phase. Phage vB_EcoS-TPF103dw was added at a multiplicity of infection (MOI) of 0.01 in the presence of CaCl₂ (final concentration 10 mM), and the mixture was incubated for 10 min at 37 °C to allow phage adsorption. This 10 min adsorption for *E. coli* one-step growth assays is documented in multiple studies (Guo et al., 2021; Necel et al., 2020; Magar et al., 2025). The phage-bacterial mixture was then centrifuged at 10,000 × g for 5 min at 4 °C. The resulting pellet was washed three times with 2 mL TSB to remove unadsorbed phages and resuspended in 20 mL of fresh TSB.

A 0.3 mL aliquot of this suspension was transferred to 29.7 mL of fresh TSB and incubated at 37 °C with shaking at 90 rpm. To determine phage titers, 1 mL samples were collected and filtered through 0.22 μm syringe filters to remove bacterial cells. The time points were designated every 10 min for 120 min time frame. The filtrates were serially diluted in SM buffer, and plaque assays were performed using the double-layer agar method, mixing 50 μL of each dilution with 100 μL of overnight ATCC 13706 and 5 mL of molten 50% TSA. Plates consisted of a 1X TSA base layer (10 mL) overlaid with 5 mL of semi-solid “50% TSA” (0.5X) top layer prepared by dissolving half the standard TSA powder in the full water volume. The number of free phages at each time point was determined by incubating the plates at 37 °C overnight. This experiment was conducted in triplicate to determine the latent period and burst size of phage vB_EcoS-TPF103dw.

### 2.6 Transmission electron microscopy

The phage purified by CsCl gradient was prepared for transmission electron microscopy (TEM) analysis (FEI Tecnai G2) using negative staining. Briefly, 5 μL of the purified phage suspension was applied to a discharged 300-mesh carbon/formvar-coated grid and allowed to adsorb for 2 min. The grid was then washed with distilled water to remove unbound material, and excess liquid was gently blotted away. Next, 5 μL of 1% uranyl acetate was applied for negative staining. After air drying, the sample was examined under the TEM.

### 2.7 Stability test

Phage stability across a range of pH values was evaluated following established methodologies with minor modifications. 1 mL of SM buffer phage at approximately 9.6 × 10^9^ log PFU/mL was mixed with 10 mL of sterile SM buffer adjusted to final pH values of 3.0, 5.0, 7.0, 10 and 12. The samples were incubated at 25 °C for 24 h. After incubation, viable phage particles were enumerated using the double-layer plaque assay. Experiments were conducted in triplicate.

For the temperature stability evaluation, phage lysate was diluted in SM buffer at a ratio of 1:9 (v/v). Aliquots of 1 mL of the diluted phage suspension were dispensed into sterile microcentrifuge tubes and incubated at temperatures of 25 °C (control), 40 °C, 50 °C, 60 °C, and 70 °C for 24 h using temperature-controlled water baths. Following incubation, phage viability was assessed using the double-layer plaque assay. Experiments were performed in quadruplicate.

### 2.8 Host range and efficiency of plating

To evaluate the host range of phage vB_EcoS-TPF103dw, a spot test assay was performed against a panel of bacterial strains (Table 1), including non-pathogenic *E. coli*, STEC, *Salmonella,* and *Listeria monocytogenes*, following previously established protocols with slight modifications (Liao et al., 2022a; Zhang et al., 2021a,b). Bacterial strains showing susceptibility in the spot assay were selected for further analysis of productive infection using efficiency of plating (EOP).

**Table 1:** Efficacy of plating (EOP).

Strain	Strain Ref No.	EOP
Generic *E. coli*	ATCC 13706 (Host)	1
STEC Non-O157	O103:H11 BAA2215	>0.1 **
	O103 RM13322	>0.1 **
	O103 H2 GG7	0.97533
	O103 H25 SJ11	0.95351
	O103:H11 SJ12	0.8852
	O26 H11:HH8	>0.1 **
	O26:H11 SJ1	>0.1 **
	O26:H2 JB285	R
	O26:11 SJZ	>0.1 **
	O26 BAA-2196	>0.1 **
	O45 SJ7	R
	O111 (RM11765)	R
	O111 (RM14488)	R
	O45-2	R
	O121:H19 (BAA-2219)	R
	O26:H11 SJ7	R
	O121-2 (RM8088)	R
	O145 (RM13514)	R
	O145 (RM112238)	R
STEC O157	O157 (35150)	R
	O157 (RM19259)	R
	O157 (43888)	R
*Escherichia alberti*	Rm9973	R
	RM15115	R
*Salmonella*	infantis 9799	R
	typhi 14028	R
	entritidis PT30	R
*Listeria monocytogenes*	CFSAN 006121	R
	CFSAN 002285	R
	CFSAN 034257	R
	CFSAN 00078	R

Several *E. coli* O103 and O26 strains supported productive phage replication, with EOP values ranging from >0.1 to high (>0.9), indicating these strains are permissive to infection. In contrast, the majority of *Salmonella*, *Listeria monocytogenes*, and other STEC strains showed no visible plaques and were classified as resistant (R). These findings suggest that the phage exhibits a narrow host range, with infectivity and replication primarily limited to specific *E. coli* serotypes.

Fresh overnight cultures of each test strain and the primary host strain ATCC 13706 were prepared in Tryptic Soy Broth (TSB) (Becton Dickinson, Sparks, MD, United States) and incubated at 37 °C for 18 h. Phage lysates were serially diluted from 10^−3^ to 10^−7^, and the titers were determined using a double-layer agar plaque assay. Plates were incubated at 37 °C overnight. The experiment was conducted in triplicate.

EOP was calculated by dividing the average plaque-forming units (PFU) on each test strain by the average PFU on the primary host strain ATCC 13706. An EOP value of 0.5 or greater was classified as high phage production efficiency, indicating robust replication on the test host. EOP values between 0.1 and 0.5 were considered medium efficiency, reflecting moderate levels of productive infection.

### 2.9 Proteomic analysis

Proteomic analyses were performed as previously described (Liao et al., 2019). CsCl-purified phage particles were reduced in Laemmli buffer containing 0.5% (v/v) 2-mercaptoethanol (Bio-Rad, Hercules, CA, United States). Samples were separated on a 1-D 12% TGX SDS-PAGE gel (Bio-Rad) and visualized with Imperial™ Protein Stain (ThermoFisher, Waltham, MA, United States). Distinct gel bands were excised, transferred to a reaction tray, and digested in-gel with sequencing-grade trypsin (Promega, Madison, WI, United States) using a DigestPro automation platform (Intavis, Köln, Germany). Peptide mixtures were analyzed by nanoflow reverse-phase liquid chromatography on an Eksigent NanoLC (Sciex, Framingham, MA, United States) equipped with a Picochip column (New Objectives, Woburn, MA, United States). Data-dependent tandem mass spectrometry was performed in positive-ion mode on an Orbitrap Elite instrument (Thermo Fisher Scientific, Waltham, MA, United States) with survey scans from m/z 200–2000 m/z; the most intense precursor ions were selected for CID fragmentation. Raw files were converted to .mgf using MS Convert (ProteoWizard) and searched with Mascot (Matrix Science, Boston, MA, United States) against a custom database containing all NCBI phage/viral proteins plus in-house phage gene translations. Search tolerances were set to 10 ppm for parent ions.

### 2.10 Antimicrobial activity test in tryptic soy broth

A phage challenge study with vB_EcoS-TPF103dw was conducted using five *E. coli* O103 isolates (BAA-2215, RM13322, GG7, SJ11, SJ12) (Table 1) with different H antigens to evaluate susceptibility across a high multiplicity of infection (MOI) of 10,000. *E. coli* were quantified via spectrophotometry at the optical density (OD) at the wavelength of 600 nm. The assay was performed in a sterile 96-well microtiter plate, with each well containing 100 μL of TSB sterile broth, 20 μL of bacterial suspension, and 20 μL of phage solution. Plates were incubated at 25 °C for 24 h. The spectrophotometer (Spectramax m2 Model) was set to read and shake every hour for the duration of 24 h. To minimize evaporation and maintain consistent humidity, outer wells were filled with sterile water. The experiment was repeated in three replicates.

### 2.11 Biofilm study

#### 2.11.1 Biofilm formation

A 10 μL loopful of *E. coli* O103:H11 SJ12 was inoculated into 10 mL of Tryptic Soy Broth (TSB) and incubated overnight at 37 °C for 12 h. Sterile stainless-steel coupons (347) Disc Coupon (0.5 in x 0.15in) from Biosurface Technologies Corporation were placed in a sterile, flat-bottom 24-well microtiter plate for biofilm formation. To minimize evaporation and prevent desiccation during incubation, the outer wells of the plate were filled with 1 mL of sterile water. From the overnight culture, 100 μL was transferred to 10 mL of fresh TSB. Subsequently, 100 μL of the diluted culture was carefully pipetted onto the surface of each coupon. Multiple coupons were prepared to account for any disruption caused by the loss of surface tension, which could result in the inoculum running off the coupon surface. Only coupons that retained the inoculum on the top surface were used in the study. Care was taken during handling and incubation setup to minimize disturbance of the inoculum.

Biofilms were incubated at 28 °C for 48 h to promote curli fiber production, which curli fiber production is inhibited at temperatures above 30 °C (Barnhart and Chapman, 2006). After biofilm formation, coupons were assigned to either a control group or a phage-treated group. Control samples were submerged in 1 mL of TSB, while the treatment group was exposed to a phage suspension at approximately 2.4 × 10^9^ PFU/mL. Both groups were incubated at 28 °C for an additional 24 h. Samples were collected at 0 and 24 h time points for enumeration.

#### 2.11.2 Phage application

Coupons were aseptically removed using sterile forceps and briefly dipped three times in sterile water to remove planktonic cells. Each coupon was placed in a 5 mL sterile tube containing 1 mL of phosphate-buffered saline (PBS), vortexed for 1 min, and then sonicated in a water bath sonicator (Fisher model CPX5800) for 10 min to detach the biofilm cells. Serial dilutions were plated in duplicate on Tryptic Soy Agar using the drop plate method for enumeration. The experiment was conducted in triplicate, and colony counts were converted to log CFU values for analysis.

#### 2.11.3 Potential depolymerase enzyme application

A phage vB_EcoS-TPF103dw lysate propagated with generic *E. coli* ATCC 13706 was subjected to a 50 kDa Amicon filtration to remove phage while retaining smaller molecular weight components. The resulting filtrate containing soluble phage-derived enzymes was applied to biofilm-coated stainless-steel coupons to assess potential anti-biofilm activity. The Amicon filtration would eliminate intact phages while permitting the passage of soluble enzymes or metabolites with biofilm-disrupting properties. Following treatment, 20 μL of the filtrate was deposited onto each coupon and incubated for 30 min before being submitted to scanning electron microscopy (SEM).

#### 2.11.4 Scanning electron microscopy

Biofilm-coated stainless-steel coupons (347) Disc Coupon (0.5 in x 0.15in) from Biosurface Technologies Corporation, were prepared for scanning electron microscopy (SEM) following standard fixation and dehydration protocols. In addition, all stainless steel coupon assays included a negative blank control, a sterile coupon with no bacteria and no phage, used to verify background sterility and instrument baselines. Samples were fixed in 3% glutaraldehyde prepared in 1 × phosphate-buffered saline (PBS) for a minimum of 2 h. Throughout all preparation steps, samples were fully submerged in solution to prevent exposure to air and subsequent desiccation artifacts. Following fixation, samples were rinsed thoroughly with PBS to remove residual fixative and then dehydrated through a graded acetone series (30, 50, 70, 95%, and three changes of 100%), with each step lasting 15 min. Coupons were transferred into filter paper envelopes and stored submerged in 100% acetone while awaiting critical point drying. Samples were dried using a critical point dryer (autosamdri-815 model) with a 15 min cycle and stored in a desiccator. Prior to imaging, dried samples were sputter-coated with platinum using a sputter coater (Denton DESK II Metal Sputter Coater) at 200 mTorr for 2 min to enhance conductivity. Samples were evaluated using a scanning electron microscope (SEM) model (JEOL JSM-7900F Schottky Field Emission Scanning Electron) to assess biofilm structure and surface morphology. SEM imaging was conducted using an accelerating voltage of 5 kV. General biofilm morphology and *E. coli* O103 cell distribution was visualized at 800x magnification. To capture phage-induced cell infection and biofilm EPS layer, higher-magnification images were obtained at ~10,000x.

### 2.12 Statistical analysis

Statistical analyses were conducted using JMP 18 software (SAS Institute Inc., Cary, NC). For experiments with multiple replicates, an ANOVA followed by Tukey’s *post hoc* test was used to compare group means and determine statistically significant differences (*p* ≤ 0.05). For TSB samples with multiple time points and treatment groups, a two-way ANOVA was performed with time and treatment as factors. Data from stainless steel coupons were analyzed using one-way ANOVA followed by Tukey’s post hoc test to evaluate differences between control and treatment groups on the same day. Reductions in STEC O103 were considered statistically significant at *p* ≤ 0.05.

## 3 Results and discussion

### 3.1 Genomic analysis

**Figure 1:**
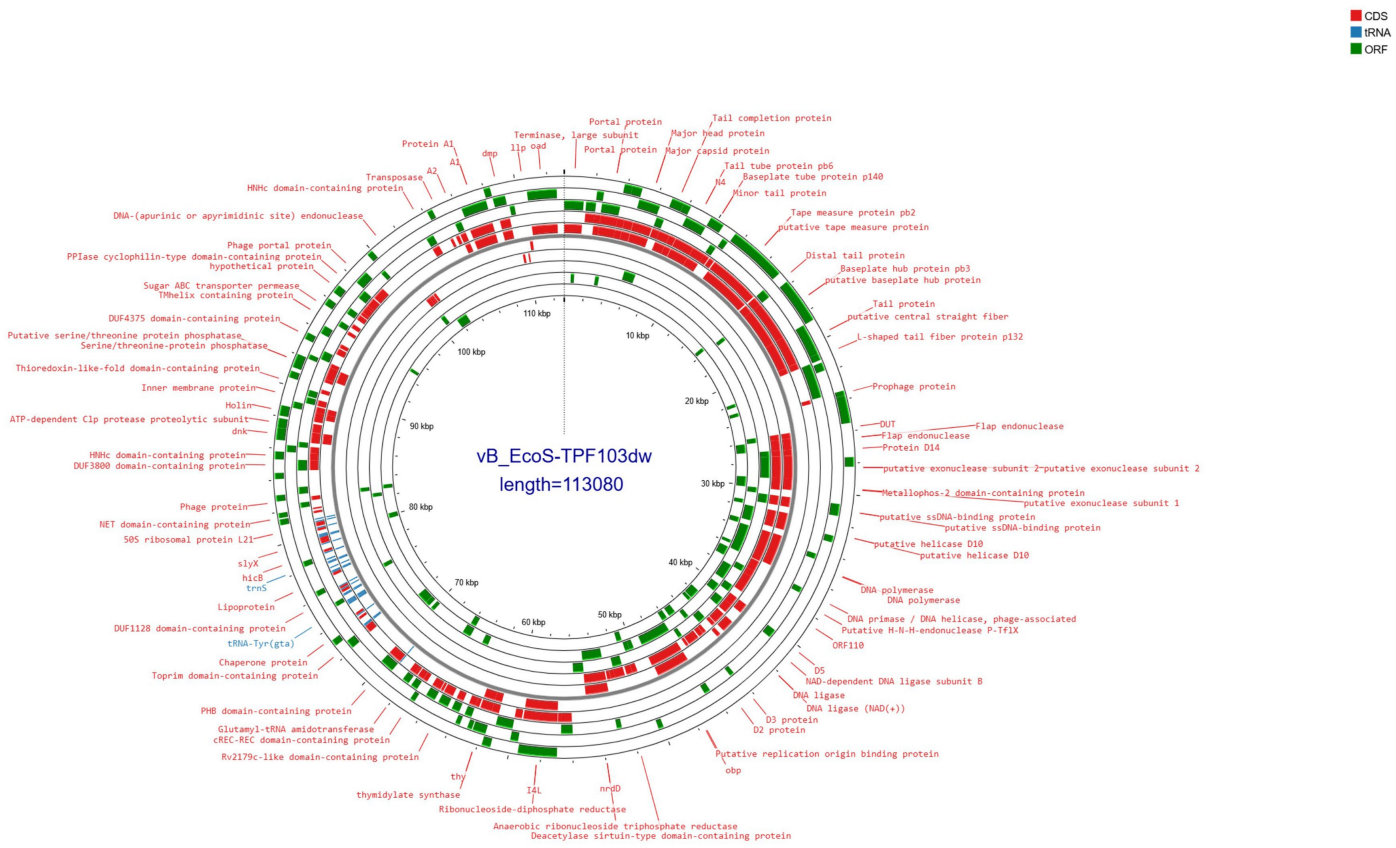
Circular genome map of *E. coli* phage vB_EcoS-TPF103dw (length = 113,080 bp), annotated using Prokka and Bakta pipelines via Proksee. Predicted coding sequences (CDS), structural RNAs (tRNA/rRNA), and open reading frames (ORFs) are represented as colored blocks (red, blue, and green, respectively) across concentric rings. Functional annotations include structural phage components, lytic enzymes (e.g., endolysins, holins), and putative host-interaction proteins. Notable features include the terminase large subunit, tail fiber proteins, and chaperones indicative of complex host recognition mechanisms.

Phage vB_EcoS-TPF103dw contained double-stranded DNA with a genome size of 113,080 bp and a GC content of 39.0% (Figure 1). The phage contained 184 open reading frames (ORFs); 119 were annotated as hypothetical proteins, whereas 41 possessed discernible functional predictions. In addition, 24 tRNA genes spanning all twenty standard amino acids, with additional isoacceptors for lysine, glutamine, methionine, and serine, were detected. The annotated genome encodes a diverse array of proteins involved in DNA replication, repair, and nucleotide metabolism, including endonucleases, exonuclease subunits, flap endonuclease, ribonucleotide reductases, primases, ligases, and deoxyuridine triphosphatase. Numerous phage structural and packaging proteins were also identified, such as the large terminase subunit, portal protein, prohead protease, major capsid protein, decoration protein, tail completion and tube proteins, distal tail and tail fiber proteins, tape measure protein, and baseplate hub components. In addition, the genome encoded proteins associated with lysis, including holins, along with several transcriptional regulators and conserved phage proteins of unknown function (Supplementary Table S2). PhageTerm analysis did not identify a definitive DNA packaging mechanism for phage vB_EcoS-TPF103dw furthermore, though ABRicate v1.0.1 (Seemann, 2016) analysis showed that phage TPF103dw did not contain any virulence, antibiotic-resistant, or lysogenic genes.

### 3.2 Comparative genomics

**Figure 2:**
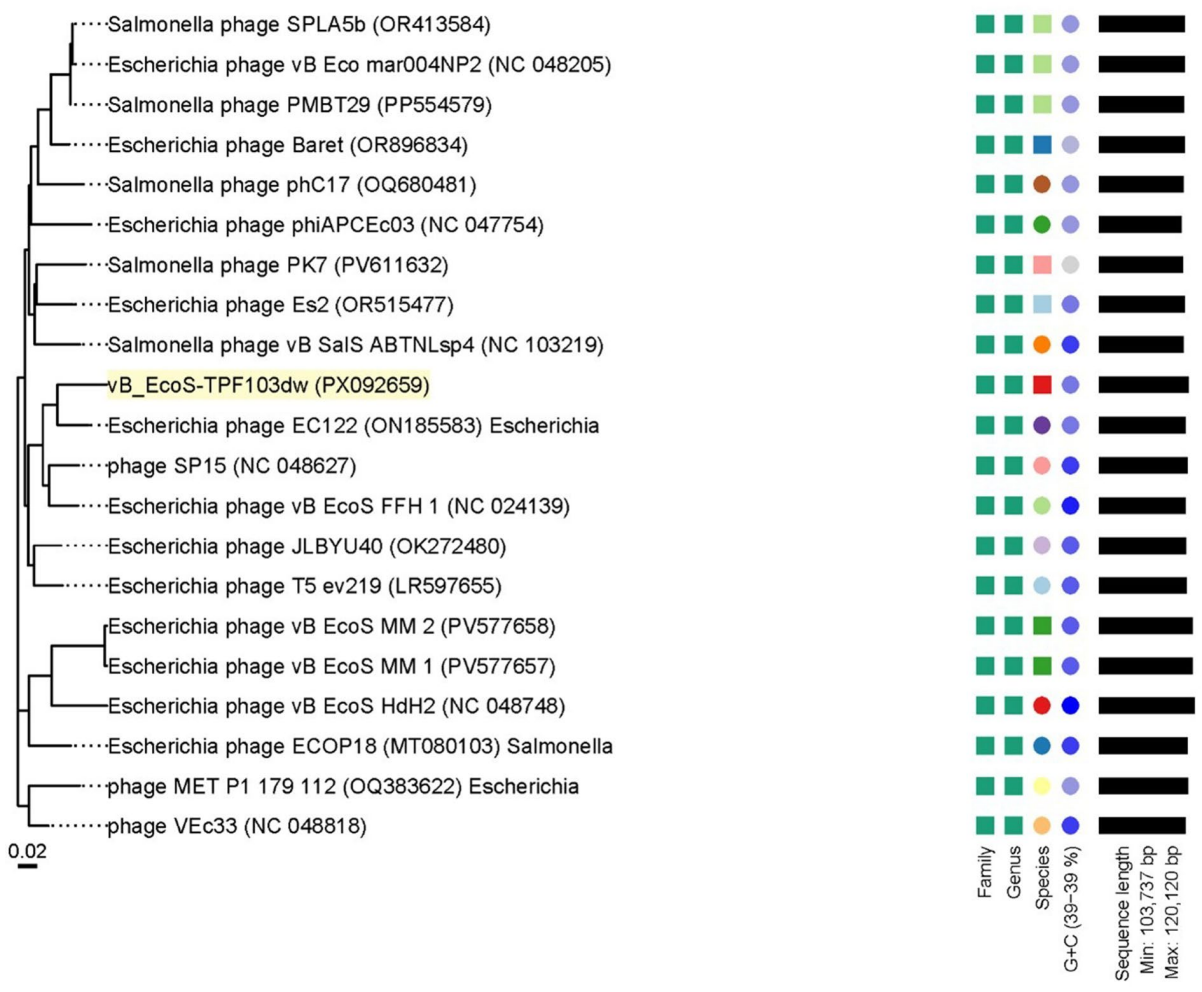
Phylogenetic analysis of whole-genome sequences of vB_EcoS-TPF103dw and close-related reference phages belonging to the *Tequintavirus* at the amino acid level using VICTOR (formula d0). Annotations, including family, genus, and species cluster proposed by VICTOR, genomic GC content, and sequence length, are given to the right-hand side of the tree.

VICTOR-based whole-genome comparison placed vB_EcoS-TPF103dw among the classic T5-like Tequintavirus (Figure 2). Within this broader group, it falls closest to *Escherichia* phage EC122. More distant branches contain T5-type phages that infect *Salmonella*, underscoring the tendency of this lineage to diversify along host lines. In addition, the pairwise comparison results indicated that vB_EcoS-TPF103dw shared an average nucleotide identity calculated based on BLAST+ (ANIb) of 92.53, 92.79, 93.10, and 93.53% with reference *Escherichia* phages T5 ev219 (LR597655), phiAPECc03 (NC_024139), SP15 (NC_048627), and JLBYU40 (OK272480), respectively. Other close relatives included *Escherichia* phage EcoS MM2 (PV577658) and EcoS MM1 (PV577657), with ANI values exceeding 92%. In each case, the aligned genome coverage ranged from 74 to 83%, surpassing the 70% coverage threshold required for genus-level classification. Consistent with the VICTOR phylogenomic tree (Figure 2), vB_EcoS-TPF103dw clustered within the T5-like phage, confirming its assignment to the *Demerecviridae* family and placement in the *Tequintavirus* genus.

**Figure 3:**
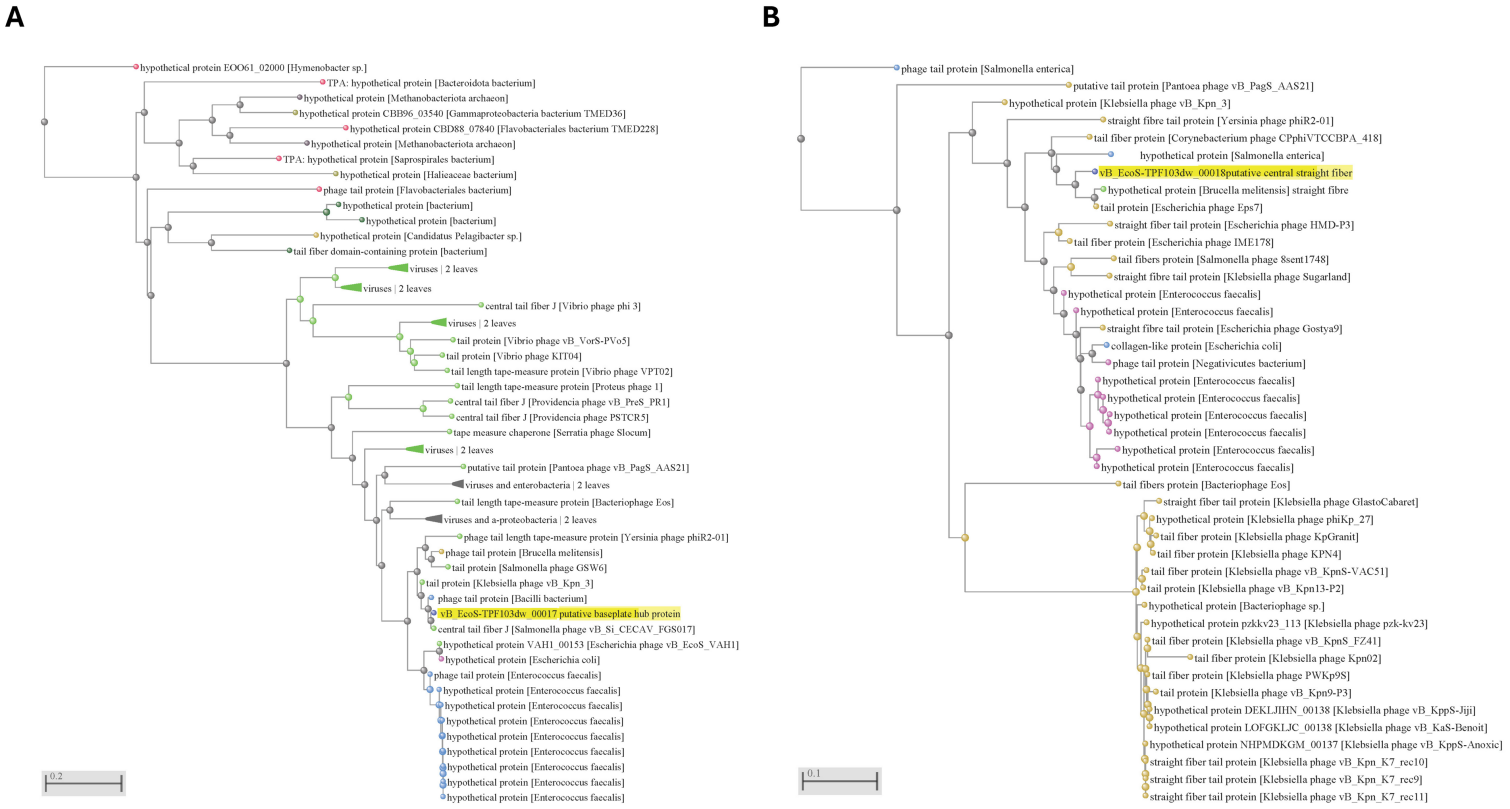
Neighbor-Joining distance trees of four putative depolymerases encoded by phage vB_EcoS-TPF103dw. **(A,B)** Correspond to protein 17 putative baseplate hub and 18 putative central straight fiber. Trees were generated by the NCBI BLASTp “Distance Tree of Results” tool using Grishin protein distances; scale bars indicate substitutions per site. The query proteins are highlighted in yellow.

Two high-probability DePP candidate hypothetical proteins were grouped with well-known structural proteins related to tail phage structures that frequently harbor depolymerase domains (Figure 3). ORF 17 aligned with the tail-fiber J and a broad tail-fiber/tape-measure branch that also contains homologues from *Vibrio*, *Proteus,* and *Providencia* phages (Figure 3A). ORF 18 showed 85.55 and 80.88% identity to the straight-fiber tail protein of *Escherichia* phage HMD-P3 and *Salmonella* phage 8sent1748 phages, respectively and clustered with other *E. coli* and *Salmonella* phages, while nearby sub-branches contained *Enterococcus*, *vibrio,* and *Klebsiella* homologues (Figure 3B).

Genomic characterization revealed two tail-associated proteins, ORFs 17 and 18, as strong depolymerase candidates. ORF 17 clustered with tail-fiber/tape-measure regions commonly linked to polysaccharide degradation, while ORF 18 aligned with straight-fiber tail proteins in *E. coli* and *Salmonella* phages, suggesting roles in host recognition. These observations suggest that vB_EcoS-TPF103dw encodes enzymatic functions capable of weakening protective barriers and improving access to embedded O103 cells.

### 3.3 Stability test

**Figure 4:**
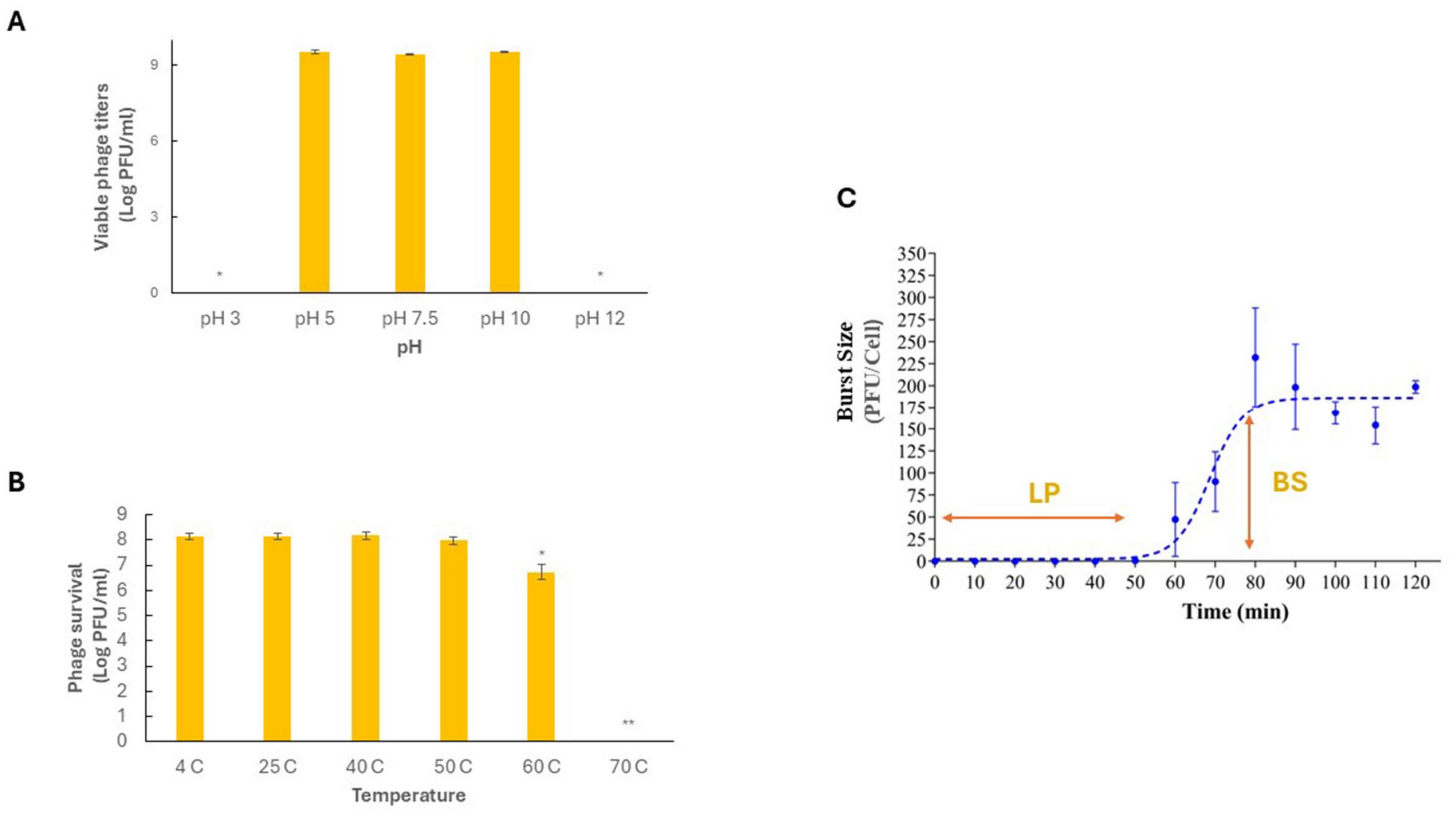
Stability of phage: biological characteristics of phage TPF103dw, including various pH stability at 25°C for 24 h **(A)**, temperature stability for 24 h **(B)**, and a one-step growth curve of generic *E. coli* ATCC 13706 **(C)**. Error bars indicate 95% confidence intervals for pH and temperature, and standard error bars for one-step growth curve. Asteriks indicated statistically significant differences among groups and *p* < 0.05.

For the pH stability test, phage titers were drastically reduced at pH 3 and pH 12, falling below the detection limit, while the phage remained stable between pH 5 and pH 10 at approximately 9.5 log PFU/mL (Figure 4A). For the thermal stability test, the phage maintained the titers of about 8 log PFU/mL between 25 °C and 50 °C without significant reduction, but titers decreased by about 1.5 log at 60 °C (*p* < 0.05). No viable phage was detected after 24 h at 70 °C, indicating complete inactivation at this temperature (Figure 4B).

### 3.4 One-step growth curve analysis and phage morphology

**Figure 5:**
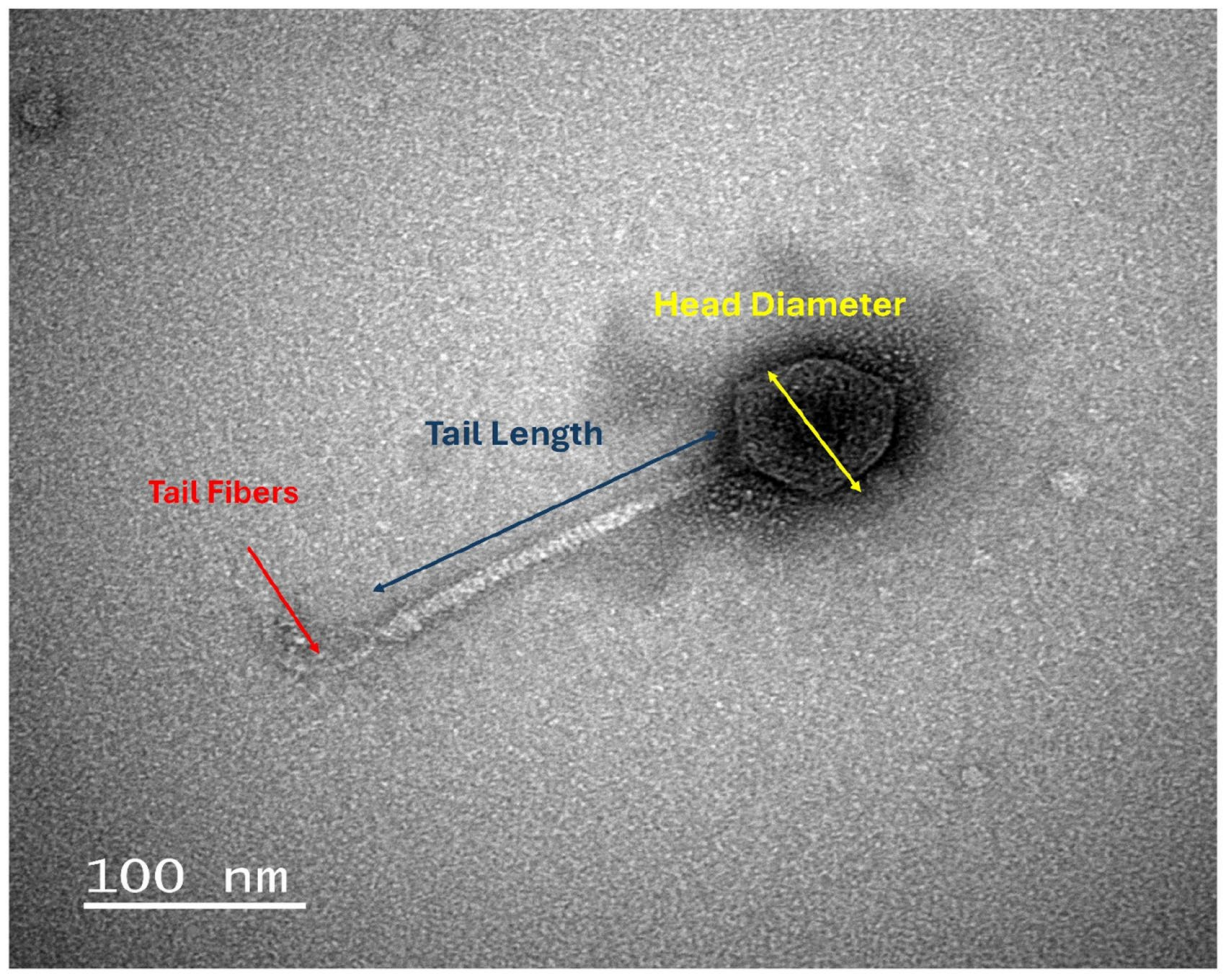
Transmission electron micrograph of phage vB_EcoS-TPF103dw. The phage shows a typical *Tequintavirus* with an icosahedral head (~65 nm) and a long, flexible, non-contractile tail (~170 nm). Image captured after CsCl purification. Scale bar = 100 nm.

The growth characteristics of phage vB_EcoS-TPF103dw, including the latent period and burst size, were evaluated. The results showed that vB_EcoS-TPF103dw had an approximately 50 min latent period (Figure 4C). An average burst size of 232 PFU per infected cell was observed at around 80 min of incubation at 37 °C. The phage exhibits an icosahedral capsid approximately 60–70 nm in diameter, attached to a long, flexible, non-contractile tail measuring roughly 150–200 nm in length (Figure 5). The tail appears striated with a clearly defined baseplate and central tail fiber.

Phage vB_EcoS-TPF103dw exhibited a burst size of 232 PFU per cell, representing a favorable trait as an antimicrobial agent, exceeding that of many previously described STEC phages (Lu and Breidt, 2015; Pinto et al., 2021; Sváb et al., 2022; Zhou et al., 2015) that have a burst size of less than 100 PFU/ml. Although the latent period of ~50 min was longer compared with latent periods found in phage UDF157lw (14 min, Liao et al., 2024) and Sa157lw (30 min, Liao et al., 2022b), this limitation was compensated by the high progeny release. The phage remained stable across a broad pH range (5 to 10) and tolerated temperatures up to 60 °C, similar to the *E. coli*-infecting phages previously isolated and used in food safety applications (Liao et al., 2022a; Liao et al., 2019; Zhang et al., 2021a,b), highlighting its robustness under processing conditions.

### 3.5 Host range and efficiency of plating

Phage vB_EcoS-TPF103dw showed productive infection primarily to a subset of *E. coli* O103 isolates, while all other tested strains were resistant (Table 1). High replication efficiency was observed on several O103 strains, including *E. coli* O103:H2 (GG7), *E. coli* O103:H25 (SJ11), and *E. coli* O103:H11 (SJ12), with EOP values exceeding 0.5. Two additional O103 strains, BAA2215 and RM13322, supported detectable plaque formation with EOP values above 0.1; however, the plaques were unusually small. In contrast, generic *E. coli* ATCC 13706, the isolation host, produced significantly larger plaques than O103 strains, while some O103 isolates formed pin-sized plaques that complicated titer enumeration (Supplementary Figure S1).

Overall, vB_EcoS-TPF103dw demonstrated a narrow host range, with productive infection restricted to select O103 and some O26 *E. coli* strains. All other STEC serogroups (O111, O45, O121, O145, and O157), *E. albertii*, *Salmonella enterica* serovars (including *Infantis, Typhi, and Enteritidis*), and *Listeria monocytogenes* strains were resistant, with no plaques observed and EOP values of zero. This host specificity suggests potential utility for targeted biocontrol applications where O103 is the primary contaminant of concern, and it could be combined with phage cocktails to broaden activity against multi-serovar STEC biofilms (O'Flynn et al., 2004).

Host range testing confirmed productive infection against several O103 isolates and select O26 strains, underscoring its utility in targeting two clinically relevant non-O157 STEC serogroups commonly associated with outbreaks. Although vB_EcoS-TPF103dw demonstrated replication on select O103 and O26 isolates, variability in lytic performance among strains highlights the necessity of cocktail approaches. The narrow host range observed here mirrors the behavior of many phages with high depolymerase specificity (Knecht et al., 2019), which may restrict efficacy against heterogeneous biofilm communities. Nonetheless, when combined with other phages encoding distinct depolymerases, vB_EcoS-TPF103dw could synergize in dismantling structurally diverse EPS matrices across multiple STEC serogroups. Such phage cocktails not only broaden host coverage but also provide functional redundancy, thereby mitigating the risk of resistance development while maximizing biofilm degradation (Naknaen et al., 2023; Molina et al., 2022; Yang et al., 2020).

### 3.6 Proteomic analysis of phage structural proteins

Seven bands related to phage vB_EcoS-TPF103dw proteins were resolved through SDS-PAGE, with apparent molecular weights ranging from approximately 15.4 to 50.7 kDa (Supplementary Figure S2). The identified bands, labeled A through F with sub-bands D-1 and D-2, included key structural proteins such as the major tail protein, major capsid protein, base-plate/tail-tube protein, head-to-tail adaptor, secondary tail protein, head maturation protease, and a putative tail protein, with amino acid sequence coverage ranging from 26 to 63% by LC–MS/MS (Table 2). All peptide matches exceeded stringent Mascot thresholds and were corroborated by BLASTp, supporting their assignment to capsid, tail, and lysis-associated functions. Importantly, all identified proteins corresponded with the genome annotation of vB_EcoS-TPF103dw.

**Table 2:** Phage proteins identified by LC–MS–MS.

Gel band	Putative function	Calculated mass kDa	Sequence coverage	No. of peptides	Mascot match	Locus BlastP match
A	Phage major tail protein	50.22	48%	13	TPF103_00011	CAL5098852
B	Phage major capsid protein	50.66	45%	21	TPF103_00007	WP_015974305
C	Base plate tail tube protein	34.62	26%	7	TPF103_00012	YP_007237112.1
D-1	Head-tail adaptor	19.25	36%	7	TPF103_00008	YP_007237116
D-2	Phage tail protein	17.29	63%	11	TPF103_00005	EFQ1274437
E	Head maturation protease	23.64	28%	6	gi_2667409259_gb_WVL05965.1_	YP_009283422.1
F	Putative tail protein	15.42	49%	5	TPF103_00019	UGO55996.1

In addition, another hypothetical protein, later aligned with a characterized endo-N-acetylneuraminidase, raises the possibility of endosialidase activity in this phage. Endosialidases are enzymes that cleave sialic acid-containing polysaccharides such as capsules, stripping away key protective barriers. Endo-N-acetylneuraminidase is a well-characterized depolymerase, and this homology provides strong corroboration of its depolymerase function (Kwiatkowski et al., 1982). Further biochemical isolation and functional validation are necessary to confirm endosialidase presence and the specificity of vB_EcoS-TPF103dw (Supplementary Figure S3).

### 3.7 *In vitro* antimicrobial activity test in broth

**Figure 6:**
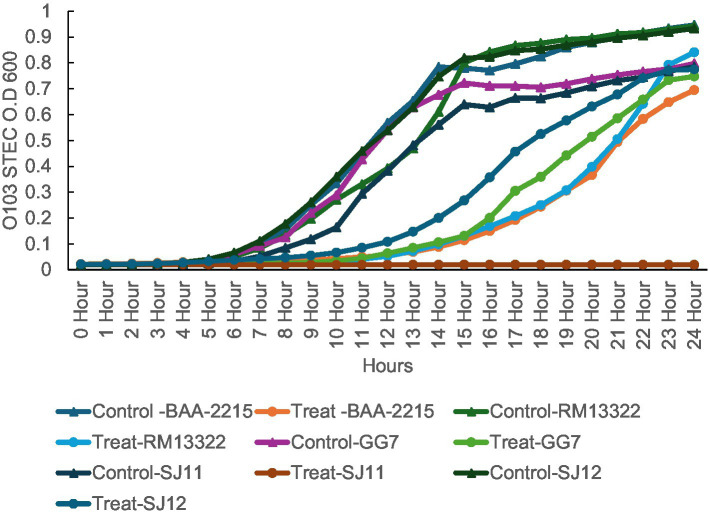
Bacterial challenge assay for *E. coli* O103, five *E. coli* O103 treated with phage TPF103dw at MOI 10,000 at 25 °C for 24 h based on the bacterial optical density at wavelength 600 nm (OD_600_). The control group only contains bacterial culture.

Phage vB_EcoS-TPF103dw demonstrated variable lytic activity across the five *E. coli* O103 isolates over 24 h (Figure 6). STEC O103:H25 (SJ11) was (*p* < 0.01) fully suppressed, showing no growth throughout the experiment. Isolates STEC O103:H2 (GG7) and STEC O103:H11 (BAA-2215) showed initial suppression with partial regrowth after 18 h, while STEC O103-1 (RM13322) displayed moderate inhibition with delayed growth compared to its control. STEC O103:H11 (SJ12) was the least affected, with only transient suppression followed by recovery to near-control levels. Control groups for all isolates grew consistently, reaching OD₆₀₀ values between 0.75 and 0.95. Statistical analysis revealed that growth suppression was significantly different from the controls at ~10 h (*p* < 0.05), although this difference was no longer significant at ~20 h, as bacterial regrowth continued. These results highlight isolate-specific susceptibility to phage treatment at high MOI.

### 3.8 Phage application against biofilm

**Figure 7:**
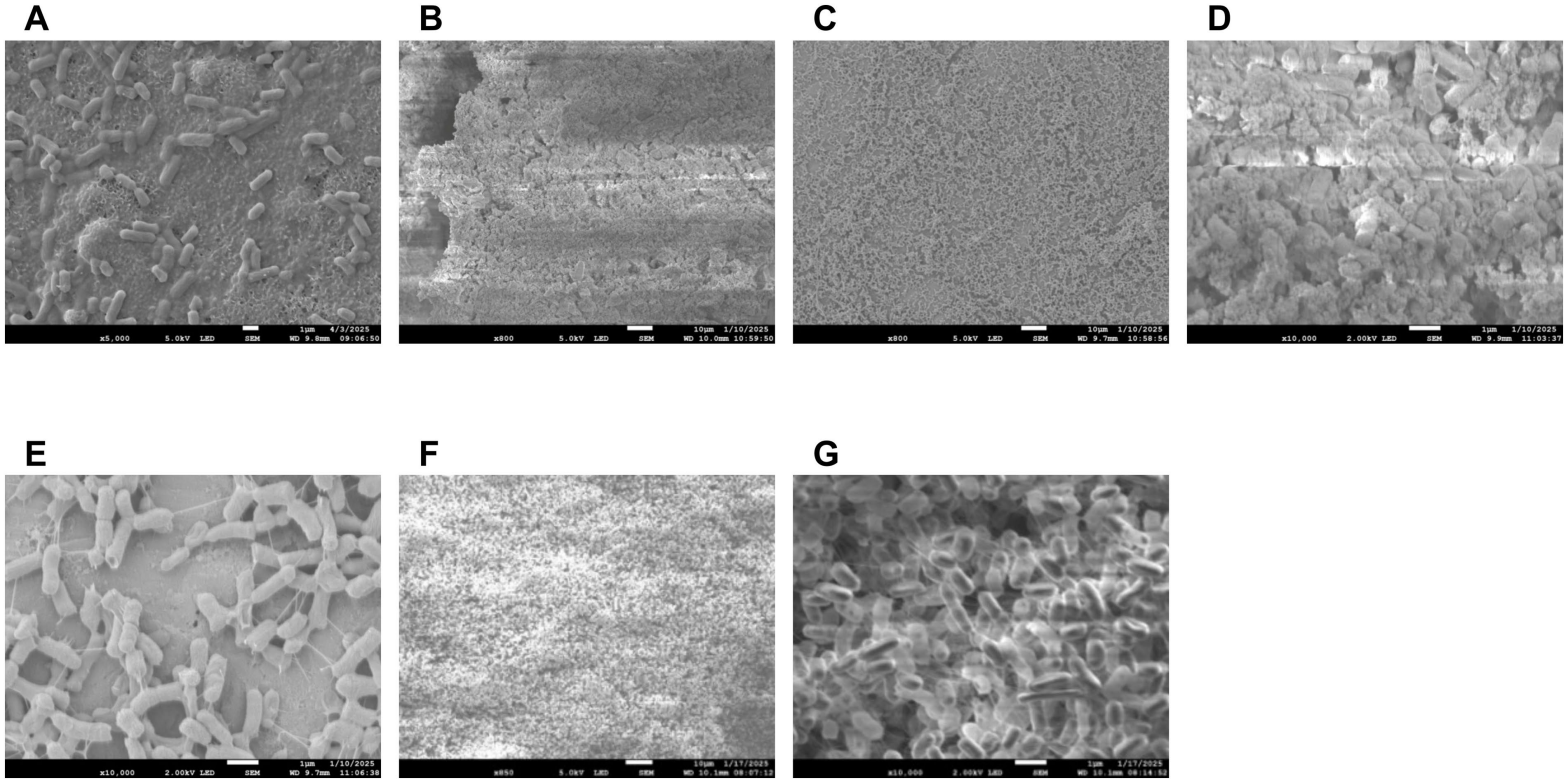
Scanning electron micrographs of *E. coli* O103 biofilms on stainless steel surfaces. Planktonic *E. coli* O103 cells were allowed to settle on sterile stainless steel coupons and visualized using SEM at 5,000 × magnification **(A)**, Day 2 untreated control biofilm at 800 × magnification, showing a thick, dense matrix with extensive extracellular material characteristic of mature biofilm **(B)**. Corresponding phage-treated biofilm at 800 × magnification, exhibiting visibly reduced biomass and a disrupted matrix structure **(C)**. Higher magnification (10,000×) view of the control biofilm, revealing dense cellular packing and abundant extracellular polymeric substances (EPS) **(D)**. Phage-treated biofilm at 10,000 × magnification, displaying isolated bacterial cells with visibly reduced EPS and disrupted aggregation **(E)**. Biofilm treated filtrate containing soluble phage-derived enzymes (850 × magnification) reveals compact cell stacking with a complete absence of visible EPS **(F)**. Biofilm treated with filtrate containing soluble phage-derived enzymes (10,000 × magnification) reveals compact cell stacking with a complete absence of visible EPS **(G)**. These images highlight phage-derived enzymatic activity that may degrade biofilm matrix, supporting potential anti-biofilm mechanisms. Images demonstrate the structural impact of phage treatment on biofilm integrity.

Planktonic cells of *E. coli* were allowed to settle onto round stainless-steel coupon discs and were subsequently visualized using scanning electron microscopy (SEM) at 5000 × magnification (Figure 7A). Unlike the dense, matrix-embedded architecture characteristic of biofilm, these cells appeared dispersed and lacked visible extracellular polymeric substance EPS (Figures 7B–E). In comparison to biofilm structure, the observed morphology confirms a non-biofilm planktonic phenotype with minimal cell-to-surface or cell-to-cell adherence, highlighting the contrast between transient surface attachment and the structured development seen in biofilm formation.

In addition, SEM was used to visualize *E. coli* O103 cells at ~20,000 × magnification, comparing healthy cells with those infected by lytic bacteriophages (Supplementary Figure S4A). Healthy O103 cells appeared intact with relatively smooth surfaces and preserved rod-shaped morphology (Supplementary Figure S2A). In contrast, phage-infected cells exhibited a highly irregular and bumpy surface, suggesting significant structural degradation (Supplementary Figure S4B). Several cells showed pronounced swelling and irregular bulges across the membrane, consistent with intracellular accumulation of phage particles. These protrusions appeared to be in the final stages of lytic cycle progression, with some cells visibly distended and on the verge of bursting, indicative of imminent phage release (Supplementary Figure S4B).

**Figure 8:**
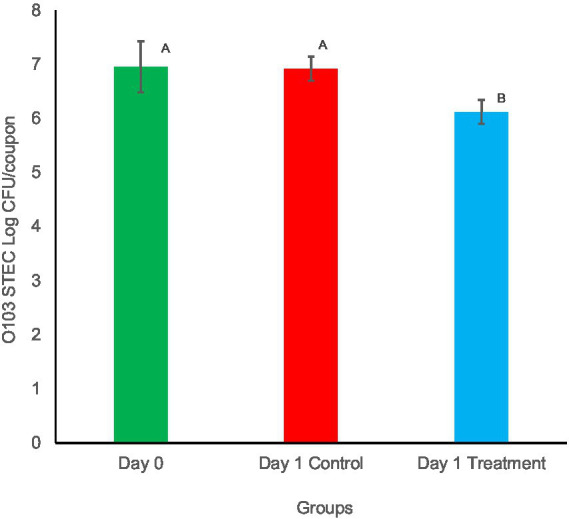
Phage disruption of biofilm on stainless steel coupon: the Y-axis represents mean STEC O103 log recovered biofilm on coupon surface after treatment with vB_EcoS-TPF103dw. Columns represent the means of STEC O103 observed from three biologically independent experiments, and error bars represent 95% confidence intervals. The X-axis represents results from analysis on the day of sample collection. Bars labeled with different superscript letters indicate statistically significant differences (*p* < 0.05), with the Day 1 treatment group showing a significant reduction compared to both the Day 0 and Day 1 control groups.

Following a 48 h biofilm formation period and 24 h exposure to either phage or control conditions, measurable differences were observed in the *E. coli* O103 biofilm populations on stainless steel coupons (Figure 8). On Day 0, the control average biofilm concentration was 6.95 log CFU/coupon. After 24 h in the control group (no phage), the biofilm remained stable, with no statistical difference (*p* > 0.05) at 6.92 log CFU/coupon. In contrast, the phage-treated group exhibited a more pronounced statistically significant (*p* < 0.05) reduction in biofilm viability, with an average of 6.12 log CFU/coupon after 24 h of exposure. Together, these results indicate strong matrix-disrupting anti-biofilm activity, evidenced by marked EPS thinning and discontinuity, accompanied by a modest immediate reduction in recoverable cells (~0.83 log10 CFU/coupon), consistent with disruption rather than complete eradication under the conditions tested (Figure 8). Several reports note minimal immediate kill, often below about 0.5 log10 (Hong et al., 2014; Mangieri et al., 2021; Tolen et al., 2018) and in some cases effectively negligible reduction, when phages are applied to STEC in biofilms or complex matrices such as beef products, produce, and cattle hides, underscoring that matrix disruption without large CFU drops can still be a meaningful outcome.

The application of bacteriophage vB_EcoS-TPF103dw disrupted the biofilm-associated *E. coli* O103 on stainless steel surfaces compared to untreated controls. While the control group showed minimal decline in biofilm viability over the 24 h period, phage treatment resulted in a nearly 1-log reduction, indicating active lytic activity against biofilm-embedded cells. This level of reduction, though modest, is notable given the protective barrier that biofilm extracellular polymeric substances (EPS) often present to antimicrobial agents (Flemming and Wingender, 2010). Phenotypic data further supported depolymerase activity. Both intact phage preparations and filtrate containing soluble phage-derived enzymes (≥50 kDa) visibly disrupted O103 biofilm matrices under SEM, producing thinner, less cohesive EPS compared to untreated controls. This biofilm reduction (approximately 1-log CFU) was modest in terms of cell killing, yet striking in terms of matrix degradation, emphasizing that the primary strength of vB_EcoS-TPF103dw lies in structural destabilization rather than eradication of embedded bacteria. These results are consistent with reports of phage-mediated biofilm disruption on abiotic surfaces (Sillankorva et al., 2012). Further optimization, such as using phage cocktails, increasing exposure time, or combining with surfactants or secondary treatments, may enhance the efficacy of biofilm disruption. Further research is needed to assess the efficacy of vB_EcoS-TPF103dw in reducing *E. coli* O103 biofilms on diverse food-contact surfaces. A key limitation of this study is that testing was restricted to a single STEC O103 strain and surface type, which does not reflect the diversity of mixed-species biofilms or the variability in surface structures that can affect depolymerase activity and phage efficacy (Flemming and Wingender, 2010).

### 3.9 Potential depolymerase enzyme activity against biofilm

In preparing vB_EcoS-TPF103dw enrichment that was filtered out phage through a 50 kda filter, it was observed under SEM that the phage-free supernatant still had depolymerase activity when tested against an O103 biofilm. Control biofilms exhibited a dense, heterogeneous structure with abundant extracellular polymeric substances (EPS) enveloping and bridging bacterial cells (Figures 7B,D). In contrast, treated biofilms with phage-free supernatant displayed cells with visibly diminished polysaccharide content (Figures 7F–G), which was not visible to the naked eye. The observed reduction in matrix material suggests the presence of phage-derived depolymerases or other enzymatic factors capable of degrading EPS, underscoring the potential of phage-associated biomolecules as anti-biofilm agents. Specifically, it was observed that a day three biofilm only treated with filtered out phage supernatant was absent of any EPS and only cells stacked upon cells were visible (Figures 7F–G).

The distinction between control and phage-treated biofilms becomes increasingly pronounced upon examination at higher magnifications 800x and 10,000x (Figure 7). At 800x magnification, the control biofilm shows a thick, continuous matrix densely packed with bacteria embedded within extensive extracellular material, indicative of a mature and structurally stable biofilm (Figures 7B,D). The phage-treated sample at the same magnification reveals a distinctly dispersed, less dense, and discontinuous biofilm, suggesting significant disruption and reduction in biofilm integrity (Figures 7C,E).

At 10,000x magnification, these differences are further highlighted: the untreated control biofilm displays extensive bacterial aggregation surrounded by dense extracellular polymeric substances (EPS), forming complex, multilayered structures indicative of healthy biofilm growth. Conversely, the phage-treated biofilm at this higher magnification clearly shows individual bacterial cells isolated from one another, accompanied by visibly reduced extracellular matrix. This suggests that the phage treatment not only disrupted bacterial aggregation but also degraded or prevented the formation of EPS, significantly impairing biofilm structural integrity and bacterial colonization. One limitation in this study utilizing SEM is that it provides high-resolution surface topology after fixation and dehydration, but is not suited for accurate volumetric or thickness measurements of hydrated EPS biofilm matrix, so we report only semi-quantitative descriptors here and quantitative EPS biovolume and thickness using confocal laser scanning microscopy with fluorescent staining and z stack analysis in future work.

The near-complete absence of EPS in both intact phage and filtrate containing soluble phage-derived enzyme treatments strongly suggests active enzymatic degradation rather than mere physical disturbance. Preliminary unpublished data indicate, by means of a gene heat map, that biofilm treated with phage is reverting to planktonic-like characteristics (Supplementary Figure S5). Through EPS degradation, vB_EcoS-TPF103dw and its associated depolymerase activity can prime biofilms for secondary treatments, making them well-suited for multi-hurdle strategies. Optimizing short phage exposures that weaken the matrix without inducing stress responses may be critical for maximizing synergy with chemical sanitizers. The predicted depolymerase activity of vB_EcoS-TPF103dw positions this phage as more than just a lytic agent; it acts as a matrix-weakening primer that can increase the susceptibility of biofilms to chemical sanitizers. For instance, in food-processing environments where chlorine or other oxidizing agents are routinely used (Chai et al., 2014; Byun et al., 2024), phage-mediated enzymatic degradation of the EPS could enhance sanitizer penetration and efficacy (Chai et al., 2014; Grygiel et al., 2024). Previous studies have shown that depolymerase-containing phages, when applied before or alongside sanitizers, enhance the efficacy of sanitizers against biofilms and can reduce the need for harsh dosing in certain settings (Chai et al., 2014; Chen et al., 2024; Mendes et al., 2024; Stachler et al., 2021). This is particularly important in industry, where minimizing sanitizer residues is critical to balancing microbial safety with food quality and regulatory compliance.

Despite encouraging genomic and proteomic evidence, several challenges remain before vB_EcoS-TPF103dw or its depolymerase enzymes can be deployed in commercial sanitation protocols. Biofilms in food production settings are rarely monospecies (Yuan et al., 2020), and their mixed microbial compositions may alter enzyme accessibility or phage adsorption (Visnapuu et al., 2022). Additionally, environmental factors such as organic load (Ly-Chatain, 2014), water hardness/ions (Jończyk et al., 2011), and surface material (Brás et al., 2024) can influence depolymerase activity and phage stability, potentially reducing consistency under real-world conditions. Addressing these variables will require testing under simulated processing conditions and exploring immobilization strategies, such as phage or enzyme coating on food-contact materials, to enhance persistence and contact efficacy.

For food processing plants challenged by recurrent STEC O103 contamination, vB_EcoS-TPF103dw presents practical potential as either a standalone biocontrol measure or as part of a multi-hurdle strategy. The application of stainless steel coupons in this study and others (Gdoura-Ben Amor et al., 2022; Jaroni et al., 2023; Wang et al., 2023) provides a proxy for industrial conveyor belts, cutting tools, and holding tanks, where biofilms are most problematic. While reductions of about 1 log may seem modest compared to chemical sanitizers, enzymatic erosion of EPS is likely to sensitize biofilms to subsequent treatments, thereby amplifying the effect of conventional interventions. Additionally, the phage’s narrow host range could be advantageous in minimizing off-target effects on beneficial microbes while specifically targeting high-risk STEC serogroups. Such properties align with the increasing industry demand for precision biocontrol tools that reduce reliance on broad-spectrum chemical disinfectants while enhancing microbial safety (Dhulipalla et al., 2025; Garvey, 2022).

## 4 Conclusion

In conclusion, this study identifies *Escherichi*a phage vB_EcoS-TPF103dw as a new *Tequintavirus* phage with high burst size, environmental stability, and depolymerase-mediated biofilm degradation. While single-strain reductions were modest, the demonstrated ability to erode protective EPS layers underscores its potential as a component of multi-hurdle sanitation strategies. Further validation across diverse STEC strains, biofilm communities, and surface materials will be essential to establish its role in food safety applications.

## Data Availability

The phage vB_EcoS-TPF103dw sequence used in this study can be found in GenBank, with the accession number PX092659.

## Footnotes

1
https://ggdc.dsmz.de/victor.php
