# Supplementary material for: Penetrating the biofilm barrier: characterization of Escherichia phage vB_EcoS-TPF103dw and harnessing depolymerase to combat Shiga toxin-producing Escherichia coli O103 biofilm
Source: Front Microbiol. 2025 Nov 27;16:1715907. doi: 10.3389/fmicb.2025.1715907 (PMC12695862; doi:10.3389/fmicb.2025.1715907)
Supplement: Supplementary file 1 [file Data_Sheet_1.PDF]

Figure S1

A. B.

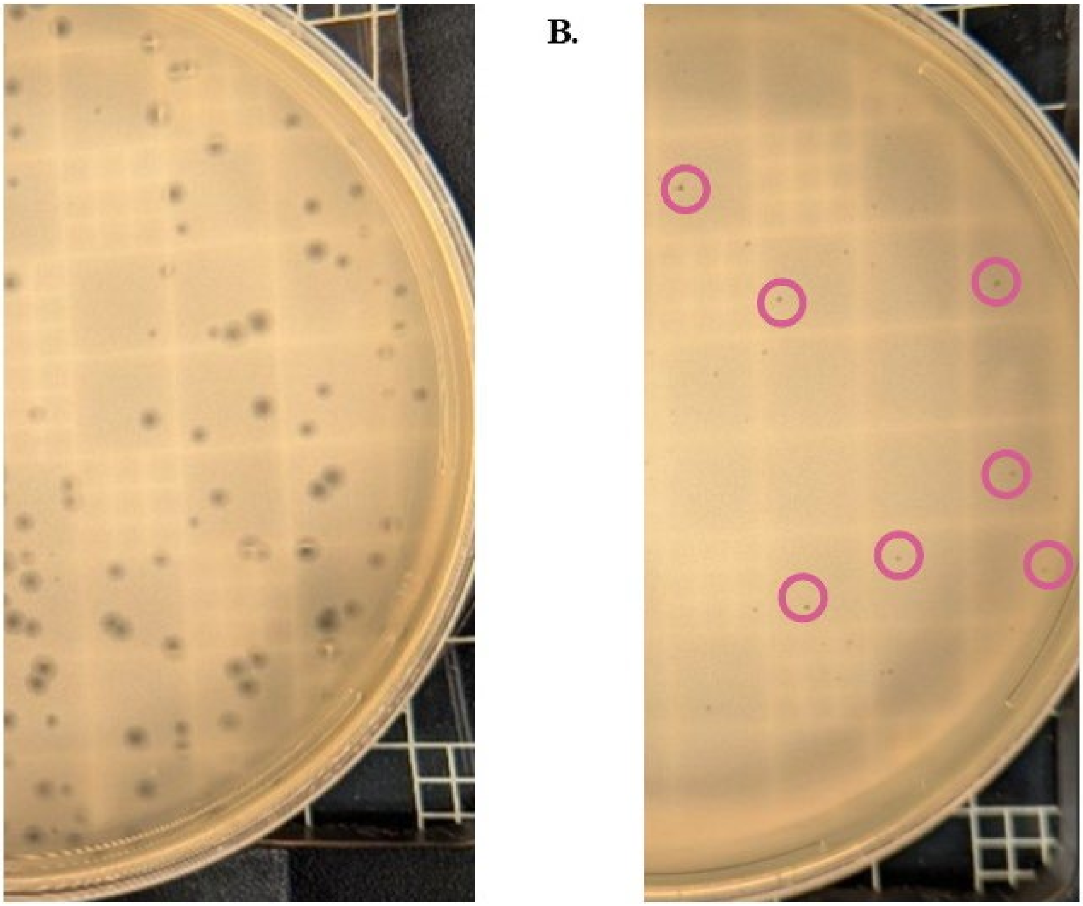

Figure S1. Phage plaque morphology on different *E. coli* hosts.

(A) Large, well-defined plaques formed on *E. coli* ATCC 13706 grown on TSA, indicating efficient lytic activity. (B) Smaller, pinpoint-sized plaques observed on *E. coli* O103 host strain ATCC 13706, suggesting a more restricted or host-specific infection profile.

These differences in plaque size may reflect host susceptibility and phage replication efficiency.

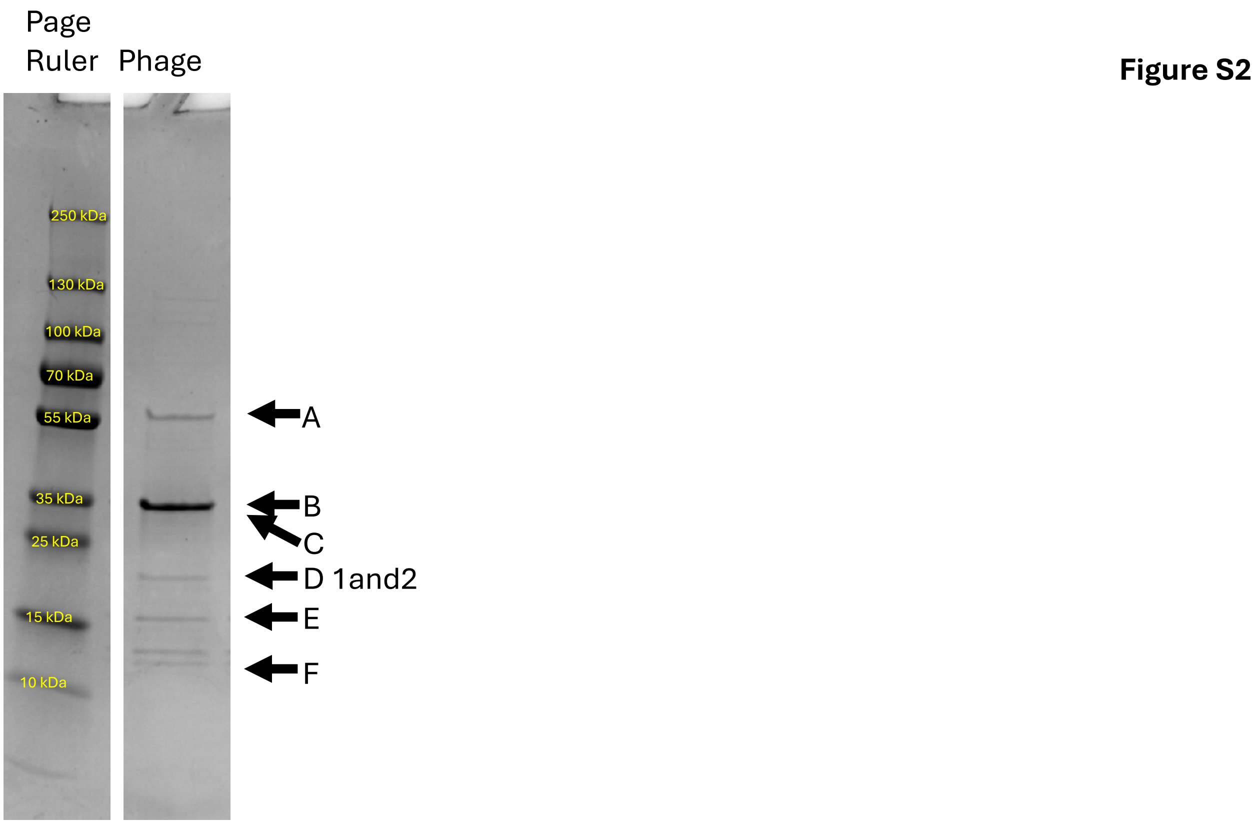

Figure S2: The proteins of phage vB_EcoS-TPF103dw detected on a 12% SDS-PAGE gel, visualized with imperial protein stain: A = Major Tail Protein; B = Major Capsid Protein; C = Base Plate Tail Tube Protein; D1 = Head-tail Adaptor; D2 = Phage Tail Protein; E = Head Maturation Protease; F = Putative Tail Protein

Figure S3

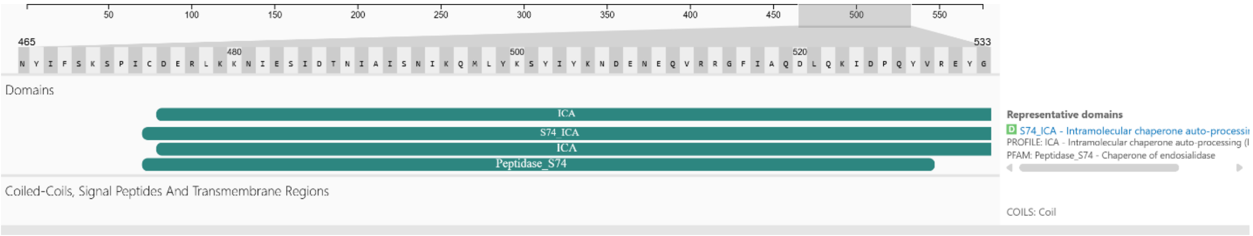

Figure S3. Domain architecture of hypothetical protein Endosialidase analyzed by InterPro. The C-terminal region (residues 465–533) harbors overlapping S74_ICA, ICA, and Peptidase_S74 domains, indicating a functional role as a chaperone for endosialidase auto-processing.

Figure S4

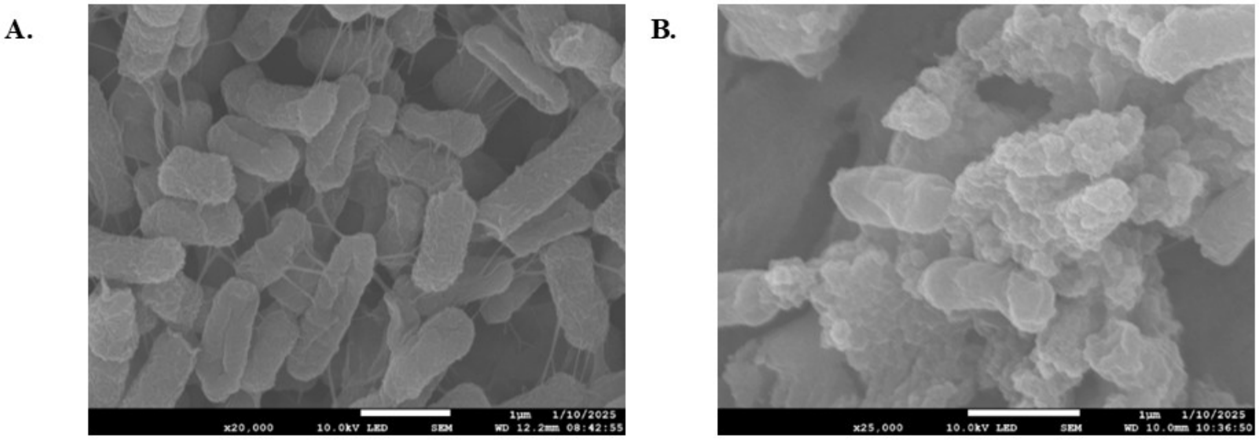

Figure S4. SEM comparison of healthy versus phage-infected *E. coli* O103 cells.
(A) Healthy control cells at 20,000× magnification exhibit smooth, intact rod-shaped morphology with visible pili-like structures. (B) Cells exposed to phage appear swollen and structurally compromised at 25,000× magnification, with irregular surface texture suggestive of lytic damage. These images demonstrate morphological changes consistent with phage infection. Scale bars = 1 µm.

Figure S5

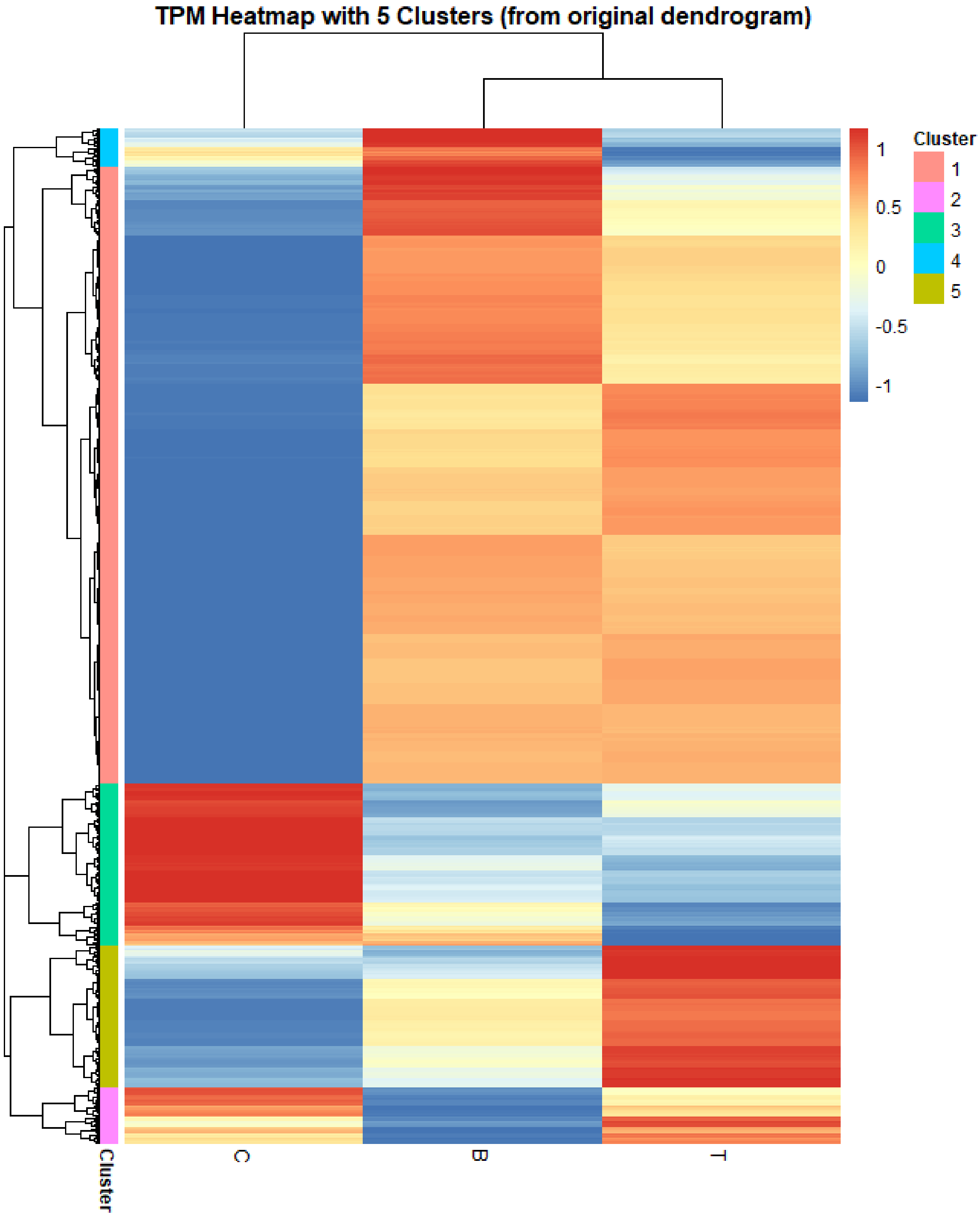

Figure S5. Exploratory research one repetition, RNA sequencing of O103 Biofilm when exposed to phage vB_EcoS-TPF103dw. (C) Control Biofilm, (B) Baseline, platonic cells (T) Biofilm Treated with phage

Figure S6

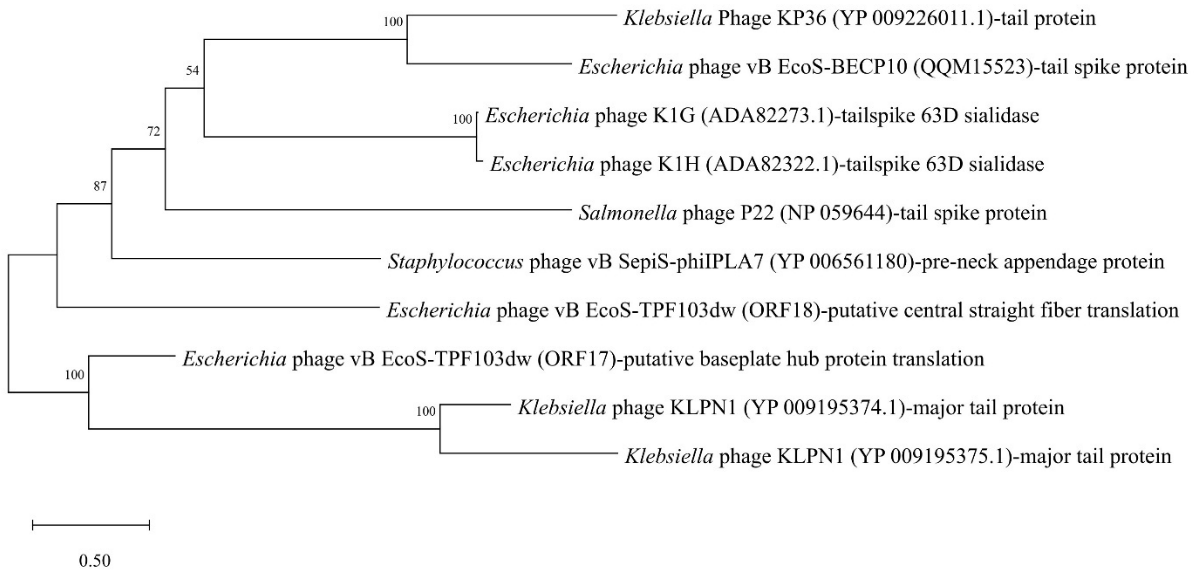

Figure S6. Maximum likelihood phylogenetic tree of ORF 17 and ORF 18, such as tail fiber and tail spike, associated with confirmed depolymerase enzyme activity, except for those from phage vB_EcoS-TPF103dw.

Table S1. DePolymerase Predictor (DePP)

Protein	Probability	Protein	Probability	Protein	Probability	Protein	Probability
1	59.40%	51	22.60%	125	18.83%	175	72.92%
2	29.98%	52	21.24%	126	9.12%	176	18.91%
3	13.78%	53	9.92%	127	19.80%	177	34.17%
4	58.82%	54	35.59%	128	6.92%	178	4.03%
5	24.70%	55	4.34%	129	19.47%	179	8.05%
6	27.92%	56	9.29%	130	19.48%	180	0.58%
7	42.34%	57	13.99%	131	7.87%	181	3.33%
8	21.01%	58	68.47%	132	8.11%	182	4.97%
9	37.22%	59	33.45%	133	35.73%	183	9.97%
10	30.08%	60	29.45%	134	24.02%	184	94.45%
11	78.42%	61	7.94%	135	17.99%		
12	61.23%	62	76.23%	136	27.50%		
13	16.77%	63	30.24%	137	23.86%		
14	10.70%	64	56.16%	138	37.18%		
15	86.80%	65	24.71%	139	15.63%		
16	37.93%	66	48.55%	140	9.29%		
17	91.81%	67	9.73%	141	9.55%		
18	91.49%	68	33.58%	142	9.78%		
19	27.02%	69	4.94%	143	16.72%		
20	82.16%	70	20.89%	144	12.95%		
21	94.00%	71	15.46%	145	40.00%		
22	17.86%	72	32.14%	146	11.60%		
23	37.22%	73	5.26%	147	18.25%		
24	21.32%	74	24.37%	148	19.40%		
25	63.33%	75	11.54%	149	8.33%		
26	40.71%	76	23.97%	150	25.19%		
27	16.85%	77	10.21%	151	6.42%		
28	37.40%	78	14.82%	152	5.70%		
29	17.29%	80	46.78%	153	15.72%		
30	61.65%	81	19.71%	154	3.19%		
31	26.65%	82	33.04%	155	4.91%		
32	69.93%	83	31.43%	156	36.97%		
33	42.01%	86	13.85%	157	14.22%		
34	32.88%	87	6.02%	158	6.97%		
35	58.68%	89	4.45%	159	28.28%		
36	35.49%	90	3.96%	160	3.47%		
37	24.49%	91	5.21%	161	37.28%		
38	32.17%	96	10.70%	162	46.80%		
39	35.42%	99	3.84%	163	3.22%		
40	11.45%	101	5.25%	164	11.07%		
41	38.62%	103	16.16%	165	7.51%		
42	14.00%	104	9.51%	166	0.67%		
43	16.04%	107	3.37%	167	12.80%		
44	15.21%	111	17.27%	168	48.03%		
45	18.10%	113	5.23%	169	34.84%		
46	6.97%	115	6.43%	170	15.10%		
47	22.61%	116	9.81%	171	4.58%		
48	79.36%	119	10.48%	172	5.81%		
49	9.44%	120	1.75%	173	16.62%		
50	14.22%	121	10.73%	174	6.90%		

Table S2. List of annotated ORFs with location and direction in TPF103dw genome.

ORF	Predicted function	Sequence (Minimum)	Sequence (Maximun)	Length	Direction
1	Terminase, large subunit CDS	1	1317	1317	forward
2	Putative endonuclease CDS	1489	2163	675	forward
3	Nicking endonuclease CDS	2153	2590	438	forward
4	Portal protein CDS	2590	3807	1218	forward
5	N5 CDS	3804	4298	495	forward
6	Prohead protease CDS	4302	4934	633	forward
7	Major capsid protein CDS	4952	6328	1377	forward
8	Head completion protein CDS	6388	6900	513	forward
9	Tail completion protein CDS	6900	7667	768	forward
10	Tail tube terminator protein CDS	7671	8156	486	forward
11	N4 CDS	8183	9577	1395	forward
12	Minor tail protein CDS	9582	10484	903	forward
13	Tape measure chaperone CDS	10477	10881	405	forward
14	Tape measure chaperone CDS	10943	11311	369	forward
15	putative tape measure protein CDS	11394	15074	3681	forward
16	Distal tail protein CDS	15184	15798	615	forward
17	putative baseplate hub protein CDS	15795	18644	2850	forward
18	putative central straight fiber CDS	18644	20701	2058	forward
19	L-shaped tail fiber protein p132 CDS	20708	21130	423	forward
20	hypothetical protein CDS	21130	23481	2352	forward
21	hypothetical protein CDS	23478	23756	279	forward

22	hypothetical protein CDS	23766	25496	1731	forward
23	DUT CDS	25558	26004	447	reverse
24	Flap endonuclease CDS	26001	26876	876	reverse
25	Protein D14 CDS	26876	27358	483	reverse
26	putative exonuclease subunit 2 CDS	27362	29200	1839	reverse
27	putative exonuclease subunit 1 CDS	29181	30122	942	reverse
28	hypothetical protein CDS	30142	30618	477	reverse
29	putative ssDNA-binding protein CDS	30656	31429	774	reverse
30	phage protein CDS	31422	31700	279	reverse
31	putative helicase D10 CDS	31927	33279	1353	reverse
32	Minor tail protein CDS	33276	33758	483	reverse
33	DNA polymerase CDS	33766	36333	2568	reverse
34	DNA primase CDS	36396	37286	891	reverse
35	Putative homing endonuclease CDS	37283	37816	534	reverse
36	DNA helicase CDS	37813	39279	1467	reverse
37	Homing endonuclease CDS	39272	39853	582	reverse
38	D5 CDS	39913	40680	768	reverse
39	DNA ligase CDS	40673	41452	780	reverse
40	DNA ligase CDS	41655	42626	972	reverse
41	Transcriptional coactivator p15 (PC4) C-terminaldomain-containing protein CDS	42693	43004	312	reverse
42	Putative endonuclease CDS	42986	43306	321	reverse
43	hypothetical protein CDS	43699	43989	291	reverse
44	D3 protein CDS	44005	44415	411	reverse
45	phage protein CDS	44519	44794	276	reverse

46	D2 protein CDS	44763	45467	705	reverse
47	phage protein CDS	45536	45769	234	reverse
48	Putative H-N-H-endonuclease P-TflIX CDS	45753	46271	519	reverse
49	obp CDS	46258	49122	2865	reverse
50	hypothetical protein CDS	49749	49943	195	reverse
51	DUF7415 domain-containing protein CDS	49933	50325	393	reverse
52	DUF7415 domain-containing protein CDS	50335	50763	429	reverse
53	RNA repair protein CDS	50766	51104	339	reverse
54	phage protein CDS	51259	51441	183	reverse
55	NAD-dependent protein deacetylase of SIR2 family CDS	51428	52255	828	reverse
56	hypothetical protein CDS	52248	52469	222	reverse
57	hypothetical protein CDS	52438	52641	204	reverse
58	phage protein CDS	52651	52932	282	reverse
59	nrdD CDS	53032	54858	1827	reverse
60	Bacteriophage T5 Orf172 DNA-binding domain-containing protein CDS	54845	55708	864	reverse
61	Putative phaosphate starvation-inducible protein CDS	56005	56757	753	forward
62	Tail length tape measure protein CDS	56759	56980	222	forward
63	I4L CDS	57018	59453	2436	forward
64	Putative H-N-H-endonuclease P-TflVIII CDS	59555	60070	516	forward
65	ribonucleoside-diphosphate reductase CDS	60079	61224	1146	forward

66	frd CDS	61221	61754	534	forward
67	thy CDS	61754	62593	840	forward
68	hypothetical protein CDS	62687	62968	282	forward
69	ribonuclease H CDS	62968	63444	477	forward
70	hypothetical protein CDS	63521	63799	279	forward
71	Virion structural protein CDS	63883	64398	516	forward
72	Metallopeptidase CDS	64440	64676	237	forward
73	Metallopeptidase CDS	64705	65406	702	forward
74	phage protein CDS	65477	65659	183	forward
75	Tail fiber protein CDS	65713	66351	639	forward
76	Cyclic-phosphate processing Receiver domain-containing protein CDS	66794	67111	318	forward
77	Cell wall hydrolase CDS	67117	67566	450	forward
78	phage protein CDS	67637	67807	171	forward
79	Recombination related exonuclease CDS	67807	68265	459	forward
80	tRNA-Arg	69090	69164	75	forward
81	Band 7 domain-containing protein CDS	69199	70146	948	forward
82	hypothetical protein CDS	70471	70887	417	reverse
83	phage protein CDS	70909	71892	984	reverse
84	DNA primase CDS	72158	72676	519	forward
85	tRNA-Ser	72698	72786	89	forward
86	tRNA-Met	72793	72867	75	forward
87	hypothetical protein CDS	72882	73283	402	forward
88	hypothetical protein CDS	73292	73477	186	forward
89	tRNA-Leu	73579	73655	77	forward
90	phage protein CDS	73671	73838	168	forward
91	phage protein CDS	73831	74037	207	forward

92	phage protein CDS	74128	74403	276	forward
93	tRNA-Tyr	74601	74688	88	forward
94	tRNA-Glu	74696	74772	77	forward
95	tRNA-Trp	74781	74857	77	forward
96	tRNA-Phe	74864	74938	75	forward
97	Homing endonuclease CDS	74956	75228	273	forward
98	tRNA-Cys	75298	75373	76	forward
99	tRNA-Asn	75381	75463	83	forward
100	phage protein CDS	75566	75751	186	forward
101	tRNA-Asp	75761	75837	77	forward
102	transposase CDS	75855	76007	153	forward
103	tRNA-Lys	76014	76089	76	forward
104	phage protein CDS	76115	76462	348	forward
105	hypothetical protein CDS	76916	77113	198	forward
106	tRNA-Pro	77123	77198	76	forward
107	tRNA-Met	77205	77282	78	forward
108	phage protein CDS	77303	77470	168	forward
109	tRNA-Lys	77472	77550	79	forward
110	tRNA-Ala	77836	77910	75	forward
111	tRNA-Leu	77916	77992	77	forward
112	hypothetical protein CDS	78215	78562	348	forward
113	tRNA-Ser	78576	78665	90	forward
114	phage protein CDS	78686	78859	174	forward
115	tRNA-His	79229	79305	77	forward
116	phage protein CDS	79331	79528	198	forward
117	hypothetical protein CDS	79521	79757	237	forward
118	tRNA-Gln	79767	79842	76	forward
119	tRNA-Gln	79849	79924	76	forward

120	phage protein CDS	80025	80276	252	forward
121	phage protein CDS	80269	80433	165	forward
122	tRNA-Thr	80444	80518	75	forward
123	hypothetical protein CDS	80593	80883	291	forward
124	tRNA-Ile	80899	80975	77	forward
125	tRNA-Met	81072	81147	76	forward
126	Putative acetyltransferase-like protein CDS	81171	81539	369	forward
127	DNA polymerase CDS	81759	81935	177	forward
128	DNA polymerase CDS	82057	82404	348	forward
129	phage protein CDS	82481	82762	282	forward
130	phage protein CDS	82755	83054	300	forward
131	phage protein CDS	83023	83466	444	forward
132	phage protein CDS	83420	83716	297	forward
133	phage protein CDS	83713	83997	285	forward
134	phage protein CDS	84108	84452	345	forward
135	phage protein CDS	84607	85305	699	forward
136	HNH nuclease domain-containing protein CDS	85302	85799	498	forward
137	I-spanin CDS	85799	86242	444	forward
138	dnk CDS	86526	87278	753	forward
139	ATP-dependent Clp protease proteolytic subunit CDS	87291	87890	600	forward
140	C1 CDS	88047	88703	657	forward
141	lys CDS	88700	89113	414	forward
142	membrane protein CDS	89191	89607	417	forward
143	phage protein CDS	89683	90114	432	forward
144	thioredoxin CDS	90107	90397	291	forward
145	DUF7167 domain-containing protein CDS	90558	90935	378	forward

146	phage protein CDS	90940	91185	246	forward
147	Serine/threonine-protein phosphatase CDS	91188	92051	864	forward
148	hypothetical protein CDS	92051	92338	288	forward
149	Serine/threonine protein phosphatase CDS	92338	92928	591	forward
150	D11 protein CDS	93096	93527	432	forward
151	phage protein CDS	93606	93884	279	forward
152	Putative homing endonuclease CDS	93878	94414	537	forward
153	Tail sheath monomer CDS	94411	94692	282	forward
154	phage protein CDS	94689	94934	246	forward
155	phage protein CDS	94924	95250	327	forward
156	Holin CDS	95247	95393	147	forward
157	Putative membrane protein CDS	95350	95550	201	forward
158	phage protein CDS	95547	96008	462	forward
159	Capsid and scaffold protein CDS	95956	96327	372	forward
160	phage protein CDS	96383	96586	204	forward
161	Homing endonuclease CDS	96546	97028	483	forward
162	phage protein CDS	97028	97213	186	forward
163	hypothetical protein CDS	97213	97824	612	forward
164	phage protein CDS	97904	98788	885	forward
165	phage protein CDS	100436	100630	195	reverse
166	DNA-(apurinic or apyrimidinic site) endonuclease CDS	100627	100971	345	reverse
167	phage protein CDS	100973	101185	213	reverse
168	Integral membrane protein CDS	101188	101337	150	reverse

169	hypothetical protein CDS	101387	101617	231	reverse
170	hypothetical protein CDS	101736	102728	993	reverse
171	Putative endonuclease CDS	103294	103812	519	forward
172	Homing endonuclease CDS	104202	104543	342	forward
173	hypothetical protein CDS	104722	104925	204	forward
174	phage protein CDS	105157	105408	252	forward
175	A2 CDS	105508	105915	408	forward
176	A1 protein CDS	105972	106169	198	forward
177	A1 CDS	106268	107932	1665	forward
178	phage protein CDS	108000	108392	393	forward
179	dmp CDS	108431	109207	777	forward
180	Putative membrane protein CDS	109372	109617	246	reverse
181	Tail assembly protein CDS	109610	109804	195	reverse
182	Tail fiber protein CDS	109801	109908	108	reverse
183	Tail fiber protein CDS	109908	110063	156	reverse
184	hypothetical protein CDS	110063	110191	129	reverse
185	llp CDS	110353	110586	234	reverse
186	oad CDS	110666	112588	1923	forward
